# Supplementary material for: Conditional generation of medical time series for extrapolation to underrepresented populations
Source: PLOS Digit Health. 2022 Jul 19;1(7):e0000074. doi: 10.1371/journal.pdig.0000074 (PMC9931259; doi:10.1371/journal.pdig.0000074)
Supplement: S1 Appendix — (PDF) [file pdig.0000074.s001.pdf]

S1 Additional Results

S1.1 On the importance of missing data

As we can see from Table A, the time series in the MIMIC-III data set are riddled with missing values. In fact, more values in a given feature sequence are missing than present. Naïvely, the first instinct may be to impute these missing entries with some value. Given missingness rates of over 90% for most features, imputation becomes less of a viable option, but as previous works have shown, the patterns of missingness in medical time series data can be highly informative [66,43].

To investigate how much information is encapsulated by the missingness patterns alone, we conduct a preliminary experiment. Using the real data[2], we first train our downstream classification task on the full set of available inputs, that is the time series of the features $\mathbf{x}_{1:T}$ and the missingness patterns $\mathbf{m}_{1:T}$. We then train the same model using only the missingness masks $\mathbf{m}_{1:T}$ and compare their evaluation scores in Table A.

Evidently, the missing value patterns are highly informative for the considered downstream classification task, as AUROC score obtained from training the model solely on the masks is only marginally below the case when the features $\mathbf{x}_{1:T}$ are added. Given the importance of the missingness masks to predict later interventions, modelling their generation deserves special attention.

Table A: AUROC scores for the `vent` classification of real data, using all features or only the missingness masks.

Input	AUROC
Real data (feats & masks)	0.811 (0.809, 0.813)
Real data (masks only)	0.796 (0.794, 0.797)

S1.2 Modelling missingness patterns

As the preliminary experiments of the preceding subsection show, the missingness patterns in the data are highly informative, meriting a deeper effort in modelling them. To this end, we compare multiple approaches to model the generative distribution of the missingess patterns $\mathbf{m}_{1:T}$.

To experimentally compare different methods to model the missing data, we train generative models with varying architectures using only the masks $\mathbf{m}_{1:T}$ as input and evaluate the usefulness of their respectively generated data for the `vent` classification task, according to the TSTR framework described in the Methods section.

[2]In the preliminary experiments in this section, we use a reduced data set consisting of 50% of the available patients and 50 out of 104 available features to enable faster iteration during development.

A first natural choice is to model the time series of missing values $\mathbf{m}_{1:T}$ as a dynamical process, given their sequential nature. We compare two approaches to model the missingness patterns as a dynamical process: first, the SRNN model [44], which represents the prototypical dynamical VAE architecture using RNNs as encoder and decoder, and second the KVAE [45], which represents a dynamical VAE approach using an internal linear state-space model to model the dynamics of the data.

Instead of modelling the missingness patterns dynamically, we also test approaches to model them as sequence-level features. That is, instead of viewing $\mathbf{m}_{1:T}$ as a time series that is generated in a recurrent fashion, we model them as static features that are generated in a single step. We implement this idea using the vanilla VAE architecture [46], where once we choose a Multi-Layer Perceptron (MLP) for the encoder and decoder, and for comparison a 1-D convolutional network to encode and decode.

We report the results of the experiments for all four architectures in Table B and can see a clear trend when comparing their performance. The first observation to be made is that modelling the missingness patterns as sequence-level static features instead of being generated by a dynamical process yields significantly better results. The second conclusion we can draw from comparing the considered approaches is that the MLP architecture seems better suited to model the missing data. As a follow up experiment, we additionally encode the feature time series $\mathbf{x}_{1:T}$ together with $\mathbf{m}_{1:T}$, to a single latent variable, and generate both from this point in the latent space. In this setting the MLP again outperforms the convolutional architecture, leading us to adapt this approach and yielding the final model architecture which we present in the Methods section.

Instead of naïvely treating the missingness patterns $\mathbf{m}_{1:T}$ and the observable features $\mathbf{x}_{1:T}$ identically and modelling them according to the same dynamical process, as the baseline models do, separating their generative processes is a key factor in generating realistic medical time series. Since two separate underlying mechanisms — the decision when to perform a measurement and the evolution of the patient's physiological state — give rise to the different time series, modelling them with separate generative processes is a natural choice. While these

Table B: Comparison of different architectures to model missingness patterns.

Architecture	Input	AUROC
MLP	masks	**0.756 (0.754, 0.758)**
Conv.	masks	0.722 (0.719, 0.725)
SRNN	masks	0.679 (0.676, 0.682)
KVAE	masks	0.515 (0.490, 0.539)
MLP	feats & masks	**0.780 (0.775, 0.784)**
Conv.	feats & masks	0.770 (0.766, 0.775)

mechanisms are distinct for each type of features, they are not independent from each other, which we capture in the connections between inferred static and dynamic latent variables in our model's architecture. Interestingly, modelling the generation of the missingness patterns via a single latent variable, instead of as a dynamical process, works better in practice. This gives the impression that the state-space models we tested are ill-suited to model the dynamics of binary variables like the missingness patterns, and perhaps specialized dynamical models would fare better.

S1.3 Fairness metric

To measure how fair the generated data of a given approach is for a downstream task, we consider the average deviation from the score obtained on the real data across all subpopulations of a given demographic. Each subpopulation is weighted equally, leading to methods that perform well for all groups obtaining higher scores than those that neglect individual groups. This metric is given by

$$m = \frac{1}{N_c} \sum_{classes}^{N_c} \frac{\text{AUROC}_{real}}{\text{AUROC}_{method}}, \tag{S1.1}$$

where N_c is the number of classes present for a demographic variable. This metric compares the similarity of downstream performance between classes, while additionally capturing the notion of improving the overall score with respect to the real data, thus quantifying the generalization performance of synthetic data across considered subpopulations. The computed metric for the visualized experiments are presented in the following Tables C and D.

Table C: Metric m for the conditional generation experiments.

	`colloid_bolus`, Ethnicity	`colloid_bolus`, Insurance	`vaso`, Age	`vaso`, Insurance
HealthGen (no cond.)	0.91 (0.89, 0.94)	1.06 (0.97, 1.15)	0.90 (0.90, 0.91)	0.92 (0.91, 0.93)
HealthGen (cond.)	1.02 (0.99, 1.05)	1.14 (1.05, 1.23)	0.92 (0.91, 0.93)	0.94 (0.93, 0.95)
SRNN	0.90 (0.88, 0.93)	0.99 (0.90, 1.08)	0.87 (0.86, 0.87)	0.84 (0.83, 0.84)
KVAE	0.87 (0.83, 0.90)	0.80 (0.69, 0.92)	0.75 (0.73, 0.79)	0.79 (0.76, 0.81)
TimeGAN	0.79 (0.75, 0.82)	0.87 (0.79, 0.94)	0.89 (0.89, 0.89)	0.91 (0.91, 0.92)

Table D: Metric m for the real data augmentation experiments.

	`colloid_bolus`, Insurance	`vaso`, Insurance
HealthGen	1.25 (1.10, 1.40)	1.03 (1.02, 1.03)
SRNN	1.11 (0.99, 1.24)	0.99 (0.98, 1.00)
KVAE	0.87 (0.74, 1.01)	1.01 (1.00, 1.02)
TimeGAN	1.04 (0.97, 1.12)	1.01 (1.00, 1.02)

S1.4 Conditional generation

Here, we present additional visualizations of settings of our conditional generation experiment, in which we utilize our model's capability to conditionally generate patient cohorts to yield data sets with an equal representation of subpopulations, ultimately increasing these data sets' fairness. These results are visualized in Fig A. Additional results are given in tabular form in the following section.

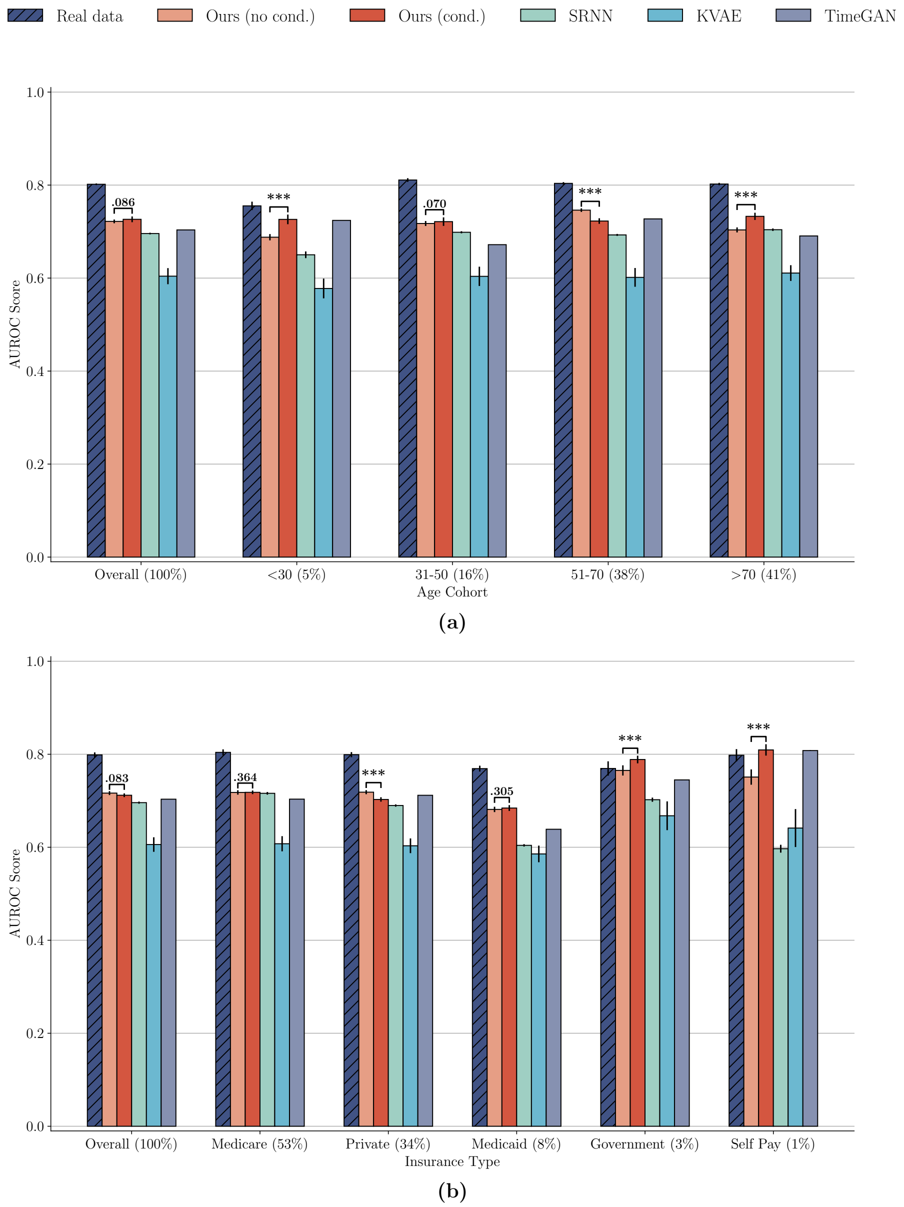

Fig A: Comparison of AUROC score between our model when conditionally and unconditionally generating synthetic data with baselines. We show these additional results of the `vaso` task for different age cohorts (a) as well for the range of insurance types (b). Significance levels between groups of interest are shown with brackets, where * corresponds to $p < 0.05$, ** to $p < 0.01$ and *** to $p < 0.001$

S1.5 Preliminary Evaluations & Additional Experiments

Here, we present an overview of the evaluation scores of the real data per subgroup and downstream task (cf. Table E). These scores constitute the starting point for experiments where we aim to improve the performance of underrepresented populations by means of conditionally generating synthetic samples of these groups with our model. As performing a full experimental run (including training and evaluation for all baseline models) is highly computationally demanding, we focus on those cases where minority groups perform significantly worse than the remaining populations. As we report in Section 2, these are indeed the cases where our model may provide a benefit via synthetic data generation and we focus on these settings in the main text. Additionally, we report all other settings for which we performed full experimental runs in the following subsections. These settings are arguably more difficult to provide a benefit for, as the difference between subgroups is not as pronounced as in those we present in the main text, or a minority group performs significantly *better* than the majority. However, in almost all cases, one would chose our model over the all other considered approaches.

S1.5.1 Real Data

Table E: Overview of the AUROC scores of the real data, per subpopulation and downstream task.

		`vent`	`vaso`	`colloid_bolus`	`crystalloid_bolus`	`niv`
	Overall	0.809 (0.807, 0.811)	0.801 (0.799, 0.803)	0.751 (0.741, 0.760)	0.613 (0.609, 0.616)	0.634 (0.632, 0.637)
Sex	Male	0.817 (0.815, 0.820)	0.793 (0.789, 0.796)	0.732 (0.722, 0.741)	0.600 (0.595, 0.604)	0.631 (0.628, 0.635)
	Female	0.798 (0.796, 0.800)	0.809 (0.806, 0.812)	0.807 (0.799, 0.816)	0.626 (0.623, 0.630)	0.645 (0.642, 0.648)
Age	<30	0.864 (0.858, 0.869)	0.755 (0.747, 0.764)	0.675 (0.643, 0.708)	0.695 (0.685, 0.704)	0.594 (0.584, 0.604)
	31-50	0.800 (0.797, 0.804)	0.811 (0.807, 0.815)	0.796 (0.785, 0.807)	0.611 (0.602, 0.620)	0.623 (0.619, 0.627)
	51-70	0.801 (0.799, 0.804)	0.804 (0.801, 0.806)	0.736 (0.721, 0.751)	0.606 (0.601, 0.611)	0.639 (0.636, 0.643)
	>70	0.813 (0.811, 0.816)	0.802 (0.800, 0.805)	0.749 (0.738, 0.759)	0.604 (0.599, 0.609)	0.639 (0.635, 0.642)
Ethnicity	White	0.808 (0.806, 0.810)	0.803 (0.800, 0.805)	0.707 (0.695, 0.720)	0.627 (0.623, 0.630)	0.639 (0.637, 0.642)
	Other	0.798 (0.795, 0.801)	0.810 (0.806, 0.814)	0.709 (0.695, 0.723)	0.588 (0.579, 0.596)	0.618 (0.614, 0.623)
	Black	0.824 (0.820, 0.829)	0.810 (0.804, 0.816)	0.757 (0.729, 0.785)	0.566 (0.555, 0.576)	0.627 (0.618, 0.636)
	Hispanic	0.822 (0.815, 0.830)	0.768 (0.758, 0.777)	0.772 (0.730, 0.813)	0.491 (0.480, 0.502)	0.638 (0.626, 0.649)
	Asian	0.843 (0.835, 0.852)	0.847 (0.839, 0.855)	-	0.547 (0.529, 0.566)	0.662 (0.651, 0.673)
Insurance	Medicare	0.809 (0.808, 0.811)	0.807 (0.805, 0.809)	0.744 (0.734, 0.753)	0.618 (0.614, 0.622)	0.635 (0.632, 0.637)
	Private	0.803 (0.801, 0.806)	0.801 (0.799, 0.804)	0.743 (0.728, 0.757)	0.598 (0.593, 0.603)	0.639 (0.636, 0.642)
	Medicaid	0.818 (0.813, 0.823)	0.770 (0.764, 0.776)	0.873 (0.857, 0.889)	0.626 (0.617, 0.635)	0.626 (0.619, 0.632)
	Government	0.826 (0.816, 0.836)	0.772 (0.757, 0.786)	0.428 (0.355, 0.501)	0.515 (0.493, 0.538)	0.613 (0.599, 0.626)
	Self Pay	0.824 (0.814, 0.834)	0.800 (0.787, 0.812)	0.824 (0.778, 0.871)	0.666 (0.650, 0.682)	0.705 (0.691, 0.720)

S1.5.2 Sex

Table F: AUROC scores for the `colloid_bolus` task.

	Overall	Male	Female
Real Data	0.751 (0.741, 0.760)	0.734 (0.722, 0.741)	0.815 (0.799, 0.816)
HealthGen (no cond.)	0.668 (0.656, 0.680)	0.653 (0.641, 0.665)	0.709 (0.690, 0.728)
HealthGen (cond.)	0.671 (0.658, 0.684)	0.660 (0.646, 0.674)	0.704 (0.684, 0.723)
SRNN	0.668 (0.662, 0.673)	0.685 (0.680, 0.690)	0.603 (0.590, 0.615)
KVAE	0.566 (0.531, 0.601)	0.557 (0.500, 0.614)	0.592 (0.547, 0.637)
TimeGAN	0.567 (0.523, 0.610)	0.601 (0.556, 0.645)	0.472 (0.426, 0.519)

S1.5.3 Age

Table G: AUROC scores for the `colloid_bolus` task.

	Overall	<30	31-50	51-70	>70
Real Data	0.751 (0.741, 0.760)	0.673 (0.643, 0.708)	0.795 (0.785, 0.807)	0.747 (0.721, 0.751)	0.749 (0.738, 0.759)
HealthGen (no cond.)	0.649 (0.631, 0.666)	0.672 (0.634, 0.710)	0.691 (0.669, 0.713)	0.637 (0.601, 0.673)	0.626 (0.593, 0.660)
HealthGen (cond.)	0.616 (0.605, 0.627)	0.618 (0.580, 0.656)	0.624 (0.608, 0.641)	0.664 (0.652, 0.676)	0.570 (0.553, 0.588)
SRNN	0.667 (0.662, 0.672)	0.657 (0.636, 0.677)	0.731 (0.723, 0.740)	0.711 (0.704, 0.718)	0.579 (0.569, 0.588)
KVAE	0.548 (0.523, 0.573)	0.510 (0.445, 0.574)	0.559 (0.524, 0.595)	0.560 (0.526, 0.595)	0.531 (0.508, 0.555)
TimeGAN	0.571 (0.558, 0.584)	0.566 (0.537, 0.594)	0.559 (0.544, 0.573)	0.613 (0.598, 0.628)	0.545 (0.532, 0.558)

Table H: AUROC scores for the `crystalloid_bolus` task.

	Overall	<30	31-50	51-70	>70
Real Data	0.613 (0.609, 0.616)	0.692 (0.685, 0.704)	0.607 (0.602, 0.620)	0.607 (0.601, 0.611)	0.603 (0.599, 0.609)
HealthGen (no cond.)	0.570 (0.568, 0.573)	0.591 (0.568, 0.615)	0.542 (0.534, 0.549)	0.571 (0.563, 0.578)	0.579 (0.579, 0.579)
HealthGen (cond.)	0.576 (0.571, 0.580)	0.612 (0.598, 0.626)	0.582 (0.575, 0.588)	0.586 (0.580, 0.591)	0.558 (0.554, 0.562)
SRNN	0.564 (0.562, 0.566)	0.628 (0.617, 0.639)	0.595 (0.590, 0.599)	0.556 (0.553, 0.558)	0.555 (0.553, 0.557)
KVAE	0.532 (0.519, 0.546)	0.523 (0.477, 0.570)	0.539 (0.523, 0.554)	0.535 (0.522, 0.549)	0.527 (0.510, 0.544)
TimeGAN	0.538 (0.532, 0.544)	0.567 (0.558, 0.576)	0.520 (0.509, 0.530)	0.539 (0.530, 0.548)	0.543 (0.541, 0.545)

S1.5.4 Ethnicity

Table I: AUROC scores for the `vaso` task.

	Overall	White	Other	Black	Hispanic	Asian
Real Data	0.801 (0.799, 0.803)	0.803 (0.800, 0.805)	0.809 (0.806, 0.814)	0.810 (0.804, 0.816)	0.770 (0.758, 0.777)	0.846 (0.839, 0.855)
HealthGen (no cond.)	0.719 (0.714, 0.724)	0.720 (0.715, 0.726)	0.717 (0.711, 0.719)	0.754 (0.746, 0.761)	0.630 (0.619, 0.640)	0.711 (0.697, 0.724)
HealthGen (cond.)	0.723 (0.720, 0.726)	0.724 (0.721, 0.728)	0.713 (0.709, 0.718)	0.759 (0.753, 0.765)	0.680 (0.673, 0.686)	0.685 (0.675, 0.695)
SRNN	0.693 (0.691, 0.696)	0.693 (0.691, 0.696)	0.687 (0.684, 0.689)	0.704 (0.698, 0.710)	0.654 (0.649, 0.658)	0.732 (0.727, 0.738)
KVAE	0.583 (0.562, 0.605)	0.587 (0.566, 0.609)	0.574 (0.552, 0.596)	0.559 (0.523, 0.595)	0.545 (0.517, 0.572)	0.636 (0.605, 0.667)
TimeGAN	0.703 (0.703, 0.704)	0.702 (0.701, 0.702)	0.704 (0.703, 0.704)	0.754 (0.754, 0.755)	0.634 (0.633, 0.634)	0.712 (0.712, 0.713)

S1.5.5 Insurance

Table J: AUROC scores for the `crystalloid_bolus` task.

	Overall	Medicare	Private	Medicaid	Government	Self Pay
Real Data	0.613 (0.609, 0.616)	0.619 (0.614, 0.622)	0.601 (0.593, 0.603)	0.629 (0.617, 0.635)	0.518 (0.493, 0.538)	0.669 (0.650, 0.682)
HealthGen (no cond.)	0.569 (0.565, 0.574)	0.583 (0.578, 0.588)	0.544 (0.534, 0.553)	0.580 (0.565, 0.594)	0.565 (0.533, 0.596)	0.616 (0.599, 0.633)
HealthGen (cond.)	0.578 (0.577, 0.580)	0.587 (0.585, 0.590)	0.561 (0.557, 0.565)	0.604 (0.598, 0.610)	0.462 (0.436, 0.488)	0.657 (0.644, 0.669)
SRNN	0.563 (0.561, 0.565)	0.560 (0.557, 0.563)	0.549 (0.546, 0.552)	0.601 (0.596, 0.607)	0.562 (0.545, 0.578)	0.759 (0.747, 0.772)
KVAE	0.539 (0.532, 0.546)	0.535 (0.526, 0.543)	0.539 (0.532, 0.546)	0.566 (0.553, 0.579)	0.510 (0.481, 0.540)	0.570 (0.537, 0.602)
TimeGAN	0.545 (0.542, 0.548)	0.545 (0.542, 0.548)	0.537 (0.533, 0.540)	0.546 (0.544, 0.548)	0.544 (0.538, 0.550)	0.706 (0.697, 0.715)

Table K: AUROC scores for the `niv` task.

	Overall	Medicare	Private	Medicaid	Government	Self Pay
Real Data	0.634 (0.632, 0.637)	0.634 (0.632, 0.637)	0.639 (0.636, 0.642)	0.629 (0.619, 0.632)	0.608 (0.599, 0.626)	0.694 (0.691, 0.720)
HealthGen (no cond.)	0.565 (0.561, 0.569)	0.557 (0.553, 0.561)	0.576 (0.570, 0.582)	0.568 (0.561, 0.575)	0.553 (0.538, 0.568)	0.623 (0.604, 0.642)
HealthGen (cond.)	0.572 (0.570, 0.574)	0.562 (0.560, 0.564)	0.586 (0.584, 0.589)	0.550 (0.545, 0.554)	0.607 (0.597, 0.616)	0.644 (0.631, 0.656)
SRNN	0.554 (0.553, 0.555)	0.542 (0.541, 0.544)	0.569 (0.567, 0.570)	0.566 (0.563, 0.570)	0.571 (0.566, 0.576)	0.541 (0.533, 0.549)
KVAE	0.521 (0.508, 0.534)	0.522 (0.507, 0.537)	0.519 (0.505, 0.533)	0.531 (0.517, 0.545)	0.493 (0.473, 0.513)	0.539 (0.510, 0.568)
TimeGAN	0.530 (0.526, 0.534)	0.528 (0.523, 0.534)	0.521 (0.516, 0.527)	0.587 (0.581, 0.592)	0.496 (0.476, 0.517)	0.551 (0.535, 0.568)

S1.6 Privacy Analysis

Here we present additional samples of the nearest neighbours privacy analysis presented in Section 2.5, together with the distances of the the respective nearest neighbours to the synthetic sample.

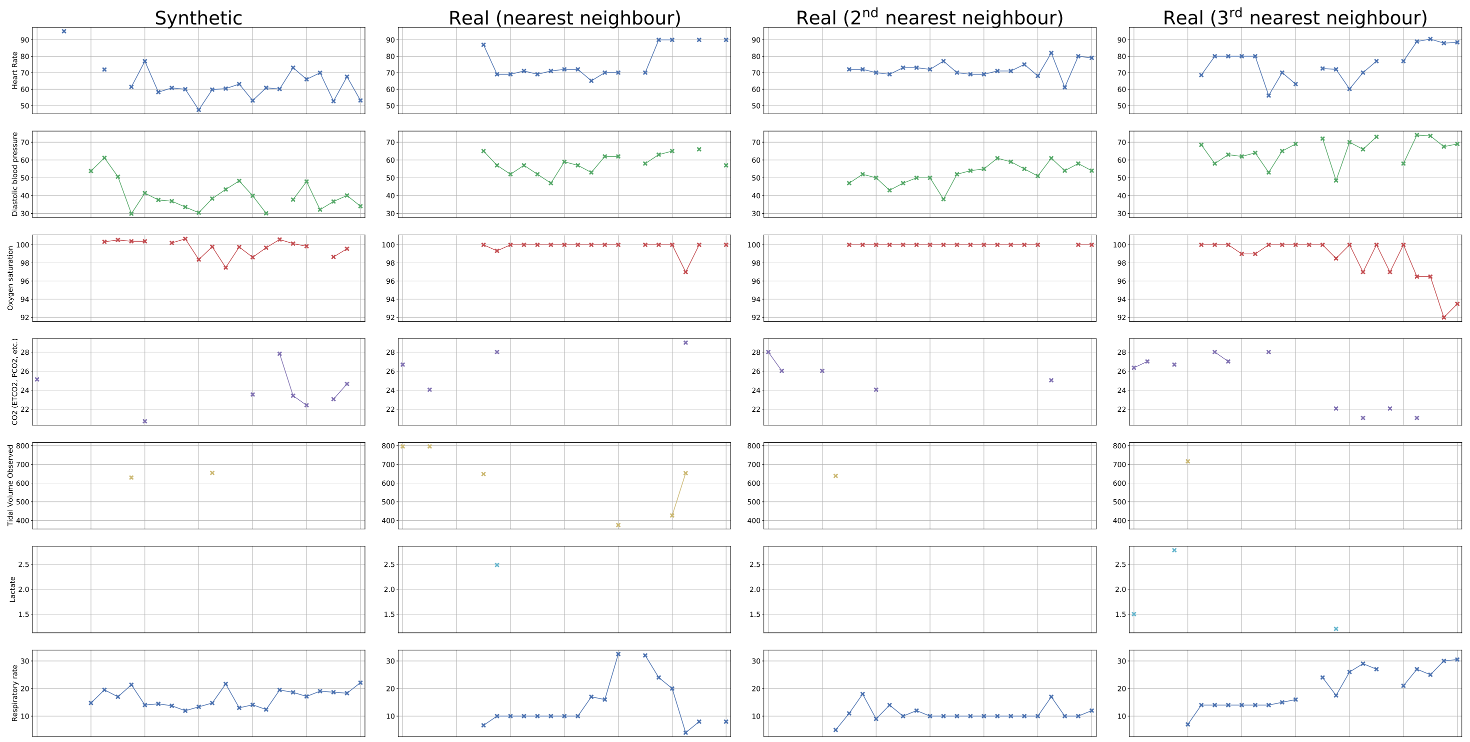

Fig B: Synthetically generated patient and its nearest real neighbours. Distances of the nearest neighbours to the synthetic sample: 5.30e-3, 5.38e-3, 5.65e-3.

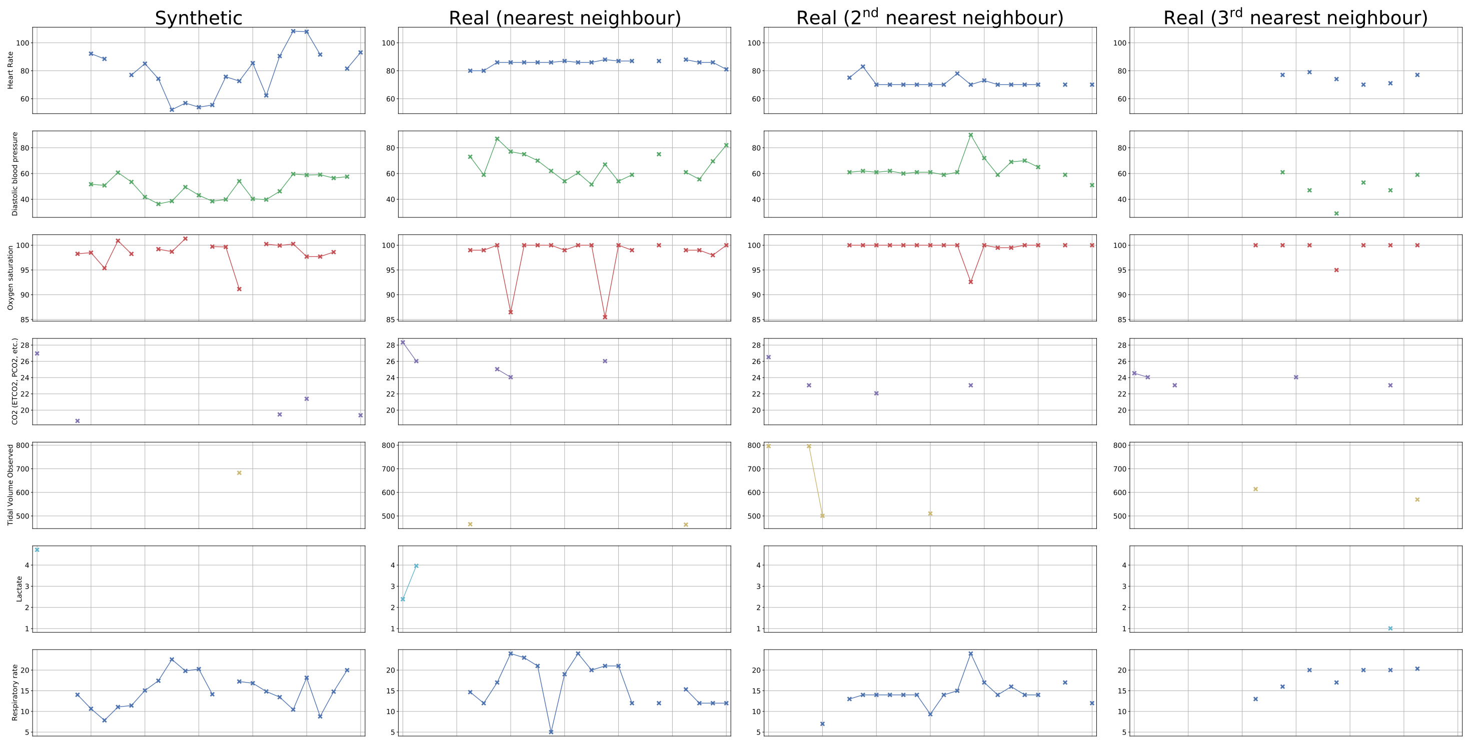

Fig C: Synthetically generated patient and its nearest real neighbours. Distances of the nearest neighbours to the synthetic sample: 16.92e-3, 17.37e-3, 18.47e-3.

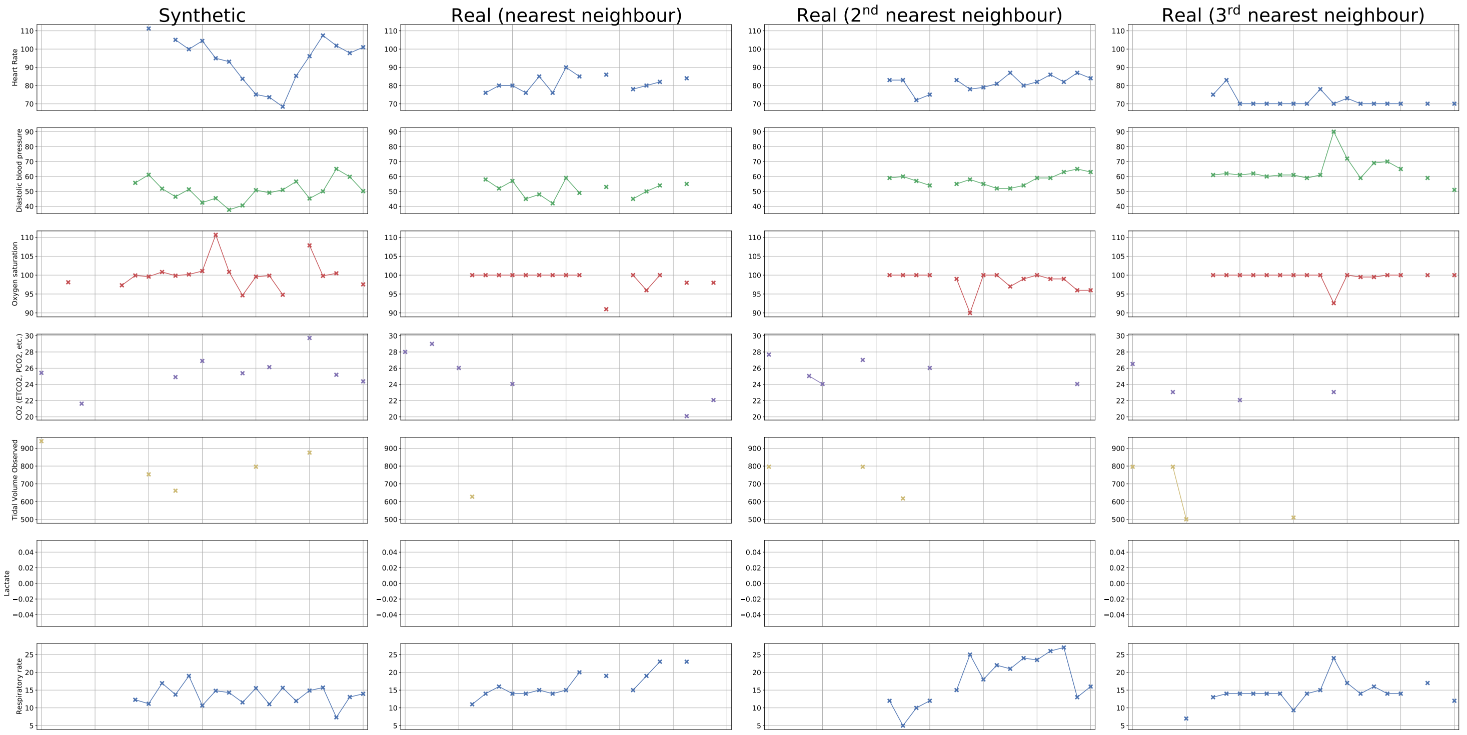

Fig D: Synthetically generated patient and its nearest real neighbours. Distances of the nearest neighbours to the synthetic sample: 20.92e-3, 29.52e-3, 29.97e-3.

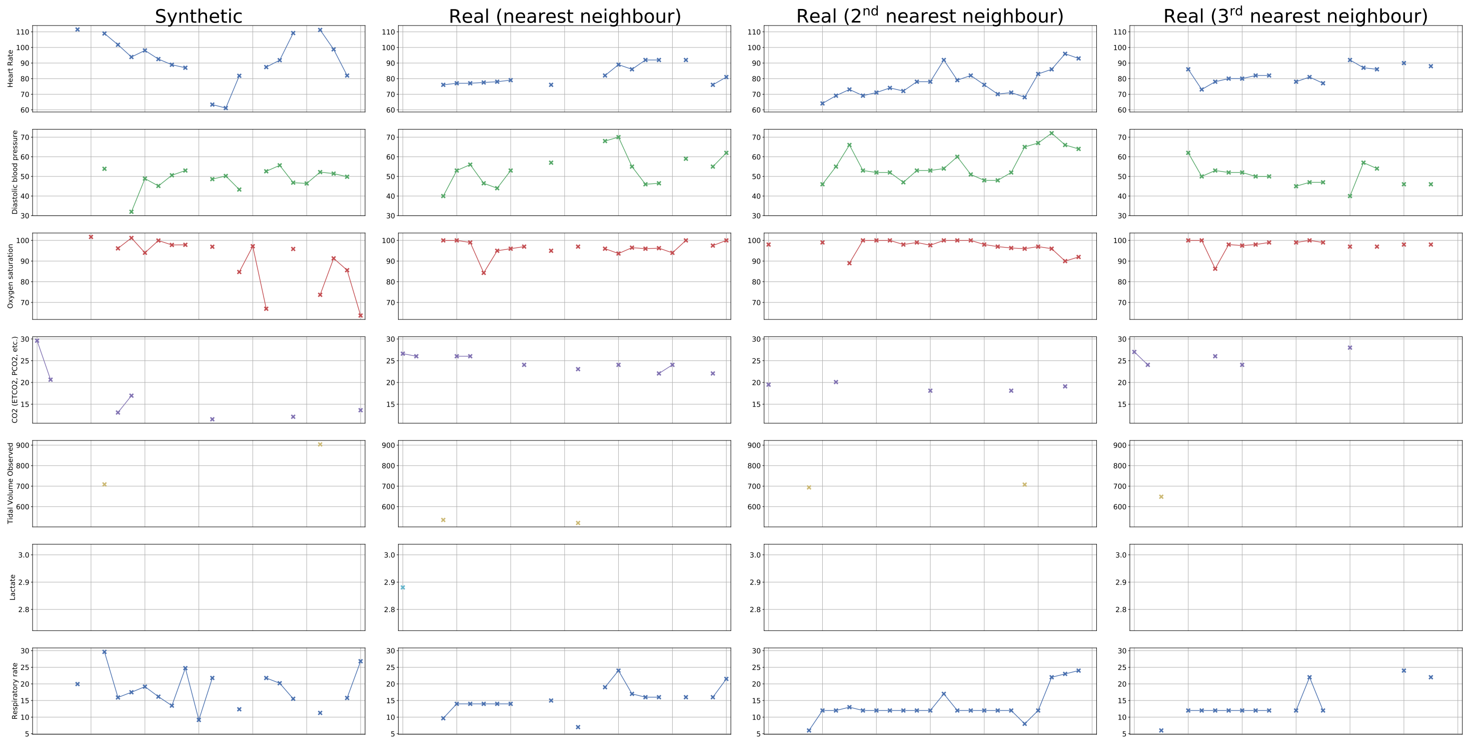

Fig E: Synthetically generated patient and its nearest real neighbours. Distances of the nearest neighbours to the synthetic sample: 36.39e-3, 36.76e-3, 38.57e-3.

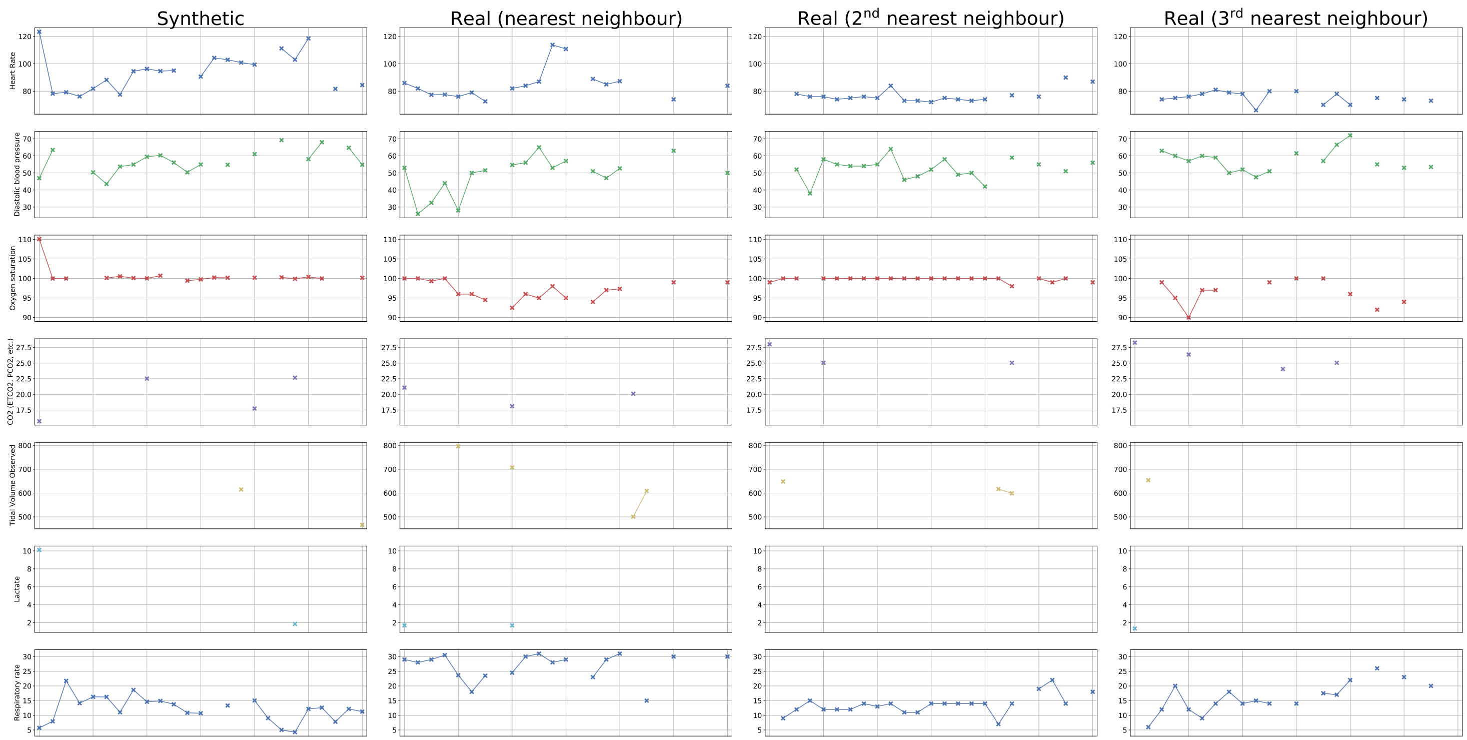

Fig F: Synthetically generated patient and its nearest real neighbours. Distances of the nearest neighbours to the synthetic sample: 19.45e-3, 19.73e-3, 20.00e-3.

S1 References

66. Razavian N, Sontag DA. Temporal Convolutional Neural Networks for Diagnosis from Lab Tests. arXiv preprint. 2015. Available from: https://arxiv.org/abs/1511.07938v4.
