# Supplementary material for: Conditional generation of medical time series for extrapolation to underrepresented populations
Source: PLOS Digit Health. 2022 Jul 19;1(7):e0000074. doi: 10.1371/journal.pdig.0000074 (PMC9931259; doi:10.1371/journal.pdig.0000074)
Supplement: S2 Appendix — (PDF) [file pdig.0000074.s002.pdf]

S2 Implementation and Training Details

Here we provide additional details on the neural architectures of our model, and on the training hyperparameters and details for all models.

S2.1 Detailed HealthGen Model Description

Generative model. The generative distribution of the missingess masks $\mathbf{m}_{1:T}$ is:

$$p_{\theta_{\mathbf{m}}}(\mathbf{m}_{1:T}|\mathbf{v},\mathbf{s},\mathbf{y}) = \prod_{t=1}^{T}\prod_{d=1}^{D}\text{Bernoulli}(m_{t,d};\mu_{t,d}), \tag{S2.1}$$

where the matrix of probabilities $\boldsymbol{\mu} \in [0,1]^{T\times D}$ is given by a DNN $d_{\mathbf{m}}(\mathbf{v},\mathbf{s},\mathbf{y})$ with a sigmoid on the last layer. The generative distribution for $\mathbf{x}_{1:T}$ is given by:

$$\mathbf{h}_1 = e_{\mathbf{h}}(\mathbf{0},\mathbf{v},\mathbf{0}), \tag{S2.2}$$

$$p_{\theta_{\mathbf{z}}}(\mathbf{z}_1|\mathbf{h}_1) = \mathcal{N}(\mathbf{z}_1;\boldsymbol{\mu}_{\theta_{\mathbf{z}}}(\mathbf{0},\mathbf{h}_1),\text{diag}\{\boldsymbol{\sigma}^2_{\theta_{\mathbf{z}}}(\mathbf{0},\mathbf{h}_1)\}), \tag{S2.3}$$

$$\mathbf{h}_t = e_{\mathbf{h}}(\mathbf{x}_{t-1},\mathbf{v},\mathbf{h}_{t-1}) \text{ for } t>1, \tag{S2.4}$$

$$p_{\theta_{\mathbf{z}}}(\mathbf{z}_t|\mathbf{z}_{t-1},\mathbf{h}_t) = \mathcal{N}(\mathbf{z}_t;\boldsymbol{\mu}_{\theta_{\mathbf{z}}}(\mathbf{z}_{t-1},\mathbf{h}_t),\text{diag}\{\boldsymbol{\sigma}^2_{\theta_{\mathbf{z}}}(\mathbf{z}_{t-1},\mathbf{h}_t)\}) \text{ for } t>1, \tag{S2.5}$$

$$p_{\theta_{\mathbf{x}}}(\mathbf{x}_t|\mathbf{z}_t,\mathbf{h}_t,\mathbf{v},\mathbf{s},\mathbf{y}) = \mathcal{N}(\mathbf{x}_t;\boldsymbol{\mu}_{\theta_{\mathbf{x}}}(\mathbf{z}_t,\mathbf{h}_t,\mathbf{v},\mathbf{s},\mathbf{y}),\text{diag}\{\boldsymbol{\sigma}^2_{\theta_{\mathbf{x}}}(\mathbf{z}_t,\mathbf{h}_t,\mathbf{v},\mathbf{s},\mathbf{y})\}). \tag{S2.6}$$

The joint distribution of all variables, conditional on the observed static features and labels, becomes:

$$\begin{aligned} p(\mathbf{x}_{1:T},&\mathbf{m}_{1:T},\mathbf{z}_{1:T},\mathbf{h}_{1:T},\mathbf{v}|\mathbf{s},\mathbf{y}) \\ &= p(\mathbf{v})p_{\theta_{\mathbf{m}}}(\mathbf{m}_{1:T}|\mathbf{v},\mathbf{s},\mathbf{y}) \\ &\prod_{t=1}^{T} p_{\theta_{\mathbf{x}}}(\mathbf{x}_t|\mathbf{z}_t,\mathbf{h}_t,\mathbf{v},\mathbf{s},\mathbf{y})p_{\theta_{\mathbf{z}}}(\mathbf{z}_t|\mathbf{z}_{t-1},\mathbf{h}_t)p_{\theta_{\mathbf{h}}}(\mathbf{h}_t|\mathbf{x}_{t-1},\mathbf{h}_{t-1},\mathbf{v}), \end{aligned} \tag{S2.7}$$

where, abusing notation, we defined:

$$p_{\theta_{\mathbf{z}}}(\mathbf{z}_1|\mathbf{z}_0,\mathbf{h}_1) = p_{\theta_{\mathbf{z}}}(\mathbf{z}_1|\mathbf{h}_1) \tag{S2.8}$$

$$p_{\theta_{\mathbf{h}}}(\mathbf{h}_1|\mathbf{x}_0,\mathbf{h}_0,\mathbf{v}) = p_{\theta_{\mathbf{h}}}(\mathbf{h}_1|\mathbf{v}) \tag{S2.9}$$

and the hidden states $\mathbf{h}_t$ are deterministic:

$$p_{\theta_{\mathbf{h}}}(\mathbf{h}_t|\mathbf{x}_{t-1},\mathbf{h}_{t-1},\mathbf{v}) = \delta(\mathbf{h}_t - \tilde{\mathbf{h}}_t) \tag{S2.10}$$

$$\tilde{\mathbf{h}}_t = \begin{cases} e_{\mathbf{h}}(\mathbf{0},\mathbf{v},\mathbf{0}) & \text{for } t=1, \\ e_{\mathbf{h}}(\mathbf{x}_{t-1},\mathbf{v},\mathbf{h}_{t-1}) & \text{for } t>1. \end{cases} \tag{S2.11}$$

Inference model. The approximate posterior distribution of $\mathbf{v}$is given by:

$$q_{\phi_{\mathbf{v}}}(\mathbf{v}|\mathbf{x}_{1:T}, \mathbf{m}_{1:T}, \mathbf{s}, \mathbf{y}) = \mathcal{N}(\mathbf{v}; \boldsymbol{\mu}_{\phi_{\mathbf{v}}}(\mathbf{x}_{1:T}, \mathbf{m}_{1:T}, \mathbf{s}, \mathbf{y}), \text{diag}\{\boldsymbol{\sigma}^2_{\phi_{\mathbf{v}}}(\mathbf{x}_{1:T}, \mathbf{m}_{1:T}, \mathbf{s}, \mathbf{y})\}), \tag{S2.12}$$

the approximate posterior of $\mathbf{z}_{1:T}$ by:

$$\mathbf{g}_T = e_{\mathbf{g}}(\mathbf{x}_T, \mathbf{h}_T, \mathbf{v}, \mathbf{0}), \tag{S2.13}$$

$$\mathbf{g}_t = e_{\mathbf{g}}(\mathbf{x}_t, \mathbf{h}_t, \mathbf{v}, \mathbf{g}_{t+1}) \text{ for } t < T, \tag{S2.14}$$

$$q_{\phi_{\mathbf{z}}}(\mathbf{z}_1|\mathbf{g}_1) = \mathcal{N}(\mathbf{z}_1; \boldsymbol{\mu}_{\phi_{\mathbf{z}}}(\mathbf{0}, \mathbf{g}_1), \text{diag}\{\boldsymbol{\sigma}^2_{\phi_{\mathbf{z}}}(\mathbf{0}, \mathbf{g}_1)\}), \tag{S2.15}$$

$$q_{\phi_{\mathbf{z}}}(\mathbf{z}_t|\mathbf{z}_{t-1}, \mathbf{g}_t) = \mathcal{N}(\mathbf{z}_t; \boldsymbol{\mu}_{\phi_{\mathbf{z}}}(\mathbf{z}_{t-1}, \mathbf{g}_t), \text{diag}\{\boldsymbol{\sigma}^2_{\phi_{\mathbf{z}}}(\mathbf{z}_{t-1}, \mathbf{g}_t)\}) \text{ for } t > 1, \tag{S2.16}$$

where $e_{\mathbf{g}}(.)$ is a backward RNN and $\mathbf{h}_t$ is the state of the forward RNN shared with the generative model. The joint inference model of HealthGen then becomes:

$$q_{\phi}(\mathbf{z}_{1:T}, \mathbf{g}_{1:T}, \mathbf{h}_{1:T}, \mathbf{v}|\mathbf{x}_{1:T}, \mathbf{m}_{1:T}, \mathbf{s}, \mathbf{y}) \tag{S2.17}$$

$$= q_{\phi_{\mathbf{v}}}(\mathbf{v}|\mathbf{x}_{1:T}, \mathbf{m}_{1:T}, \mathbf{s}, \mathbf{y}) \tag{S2.18}$$

$$\prod_{t=1}^{T} q_{\phi_{\mathbf{z}}}(\mathbf{z}_t|\mathbf{z}_{t-1}, \mathbf{g}_t) q_{\phi_{\mathbf{g}}}(\mathbf{g}_t|\mathbf{x}_t, \mathbf{h}_t, \mathbf{g}_{t+1}, \mathbf{v}) p_{\theta_{\mathbf{h}}}(\mathbf{h}_t|\mathbf{x}_{t-1}, \mathbf{h}_{t-1}, \mathbf{v}), \tag{S2.19}$$

where, abusing notation, we defined:

$$q_{\phi_{\mathbf{z}}}(\mathbf{z}_1|\mathbf{z}_0, \mathbf{g}_1) = q_{\phi_{\mathbf{z}}}(\mathbf{z}_1|\mathbf{g}_1), \tag{S2.20}$$

$$q_{\phi_{\mathbf{g}}}(\mathbf{g}_T|\mathbf{x}_T, \mathbf{h}_T, \mathbf{g}_{T+1}, \mathbf{v}) = q_{\phi_{\mathbf{g}}}(\mathbf{g}_T|\mathbf{x}_T, \mathbf{h}_T, \mathbf{v}), \tag{S2.21}$$

the hidden states $\mathbf{g}_t$ are deterministic:

$$q_{\phi_{\mathbf{g}}}(\mathbf{g}_t|\mathbf{x}_t, \mathbf{h}_t, \mathbf{g}_{t+1}, \mathbf{v}) = \delta(\mathbf{g}_t - \tilde{\mathbf{g}}_t) \tag{S2.22}$$

$$\tilde{\mathbf{g}}_t = \begin{cases} e_{\mathbf{g}}(\mathbf{x}_t, \mathbf{h}_t, \mathbf{v}, \mathbf{0}) & \text{for } t = T, \\ e_{\mathbf{g}}(\mathbf{x}_t, \mathbf{h}_t, \mathbf{v}, \mathbf{g}_{t+1}) & \text{for } t < T, \end{cases} \tag{S2.23}$$

and $p_{\theta_{\mathbf{h}}}(\mathbf{h}_t|\mathbf{x}_{t-1}, \mathbf{h}_{t-1}, \mathbf{v})$ was defined in the generative model.

Training HealthGen. The derivation of the ELBO which is maximized to train HealthGen begins by writing the lower bound of the data log likelihood as follows:

$$\log p(\mathbf{x}, \mathbf{m}|\mathbf{s}, \mathbf{y}) = \log \int_{\mathbf{z}, \mathbf{v}} p(\mathbf{x}, \mathbf{m}|\mathbf{s}, \mathbf{y}) d\mathbf{z} d\mathbf{v} \tag{S2.24}$$

$$= \log \mathbb{E}_{q(\mathbf{z}, \mathbf{v}|\mathbf{x}, \mathbf{m}, \mathbf{s}, \mathbf{y})} \left[\frac{p(\mathbf{x}, \mathbf{m}, \mathbf{z}, \mathbf{v}|\mathbf{s}, \mathbf{y})}{q(\mathbf{z}, \mathbf{v}|\mathbf{x}, \mathbf{m}, \mathbf{s}, \mathbf{y})} \right] \tag{S2.25}$$

$$\geq \mathbb{E}_{q(\mathbf{z}, \mathbf{v}|\mathbf{x}, \mathbf{m}, \mathbf{s}, \mathbf{y})} \left[\log \frac{p(\mathbf{x}, \mathbf{m}, \mathbf{z}, \mathbf{v}|\mathbf{s}, \mathbf{y})}{q(\mathbf{z}, \mathbf{v}|\mathbf{x}, \mathbf{m}, \mathbf{s}, \mathbf{y})} \right] =: \mathcal{L}(\theta, \phi) \tag{S2.26}$$

where we dropped the subscript $1{:}T$ when referring to the entire sequence. We can obtain the joint of the inference model in the denominator by marginalizing over $\mathbf{g}$ and $\mathbf{h}$, using the fact that their posteriors are deltas:

$$q(\mathbf{z},\mathbf{v}|\mathbf{x},\mathbf{m},\mathbf{s},\mathbf{y}) = \int_{\mathbf{g},\mathbf{h}} q(\mathbf{z},\mathbf{h},\mathbf{g},\mathbf{v}|\mathbf{x},\mathbf{m},\mathbf{s},\mathbf{y}) d\mathbf{g} d\mathbf{h} \tag{S2.27}$$

$$= q_{\phi_{\mathbf{v}}}(\mathbf{v}|\mathbf{x},\mathbf{m},\mathbf{s},\mathbf{y}) \prod_{t=1}^{T} q_{\phi_{\mathbf{z}}}(\mathbf{z}_t|\mathbf{z}_{t-1},\tilde{\mathbf{g}}_t), \tag{S2.28}$$

where $\tilde{\mathbf{g}}$ is the sequence of deterministic states of the backward RNN, and the states $\tilde{\mathbf{h}}$ of the forward RNN are directly used only to compute $\tilde{\mathbf{g}}$. Similarly, we can marginalize $\mathbf{h}$ in the generative model:

$$p(\mathbf{x},\mathbf{m},\mathbf{z},\mathbf{v}|\mathbf{s},\mathbf{y}) = p(\mathbf{v}) p_{\theta_{\mathbf{m}}}(\mathbf{m}|\mathbf{v},\mathbf{s},\mathbf{y}) \prod_{t=1}^{T} p_{\theta_{\mathbf{x}}}(\mathbf{x}_t|\mathbf{z}_t,\tilde{\mathbf{h}}_t,\mathbf{v},\mathbf{s},\mathbf{y}) p_{\theta_{\mathbf{z}}}(\mathbf{z}_t|\mathbf{z}_{t-1},\tilde{\mathbf{h}}_t).$$

The ELBO can finally be rewritten as follows:

$$\begin{aligned}
\mathcal{L}(\theta,\phi) = \mathbb{E}_{q(\mathbf{z},\mathbf{v}|\mathbf{x},\mathbf{m},\mathbf{s},\mathbf{y})} \Bigg[& \log p_{\theta_{\mathbf{m}}}(\mathbf{m}|\mathbf{v},\mathbf{s},\mathbf{y}) + \sum_t \log p_{\theta_{\mathbf{x}}}(\mathbf{x}_t|\mathbf{z}_t,\tilde{\mathbf{h}}_t,\mathbf{v},\mathbf{s},\mathbf{y}) \\
& + \log \frac{p(\mathbf{v})}{q_{\phi_{\mathbf{v}}}(\mathbf{v}|\mathbf{x},\mathbf{m},\mathbf{s},\mathbf{y})} + \sum_t \log \frac{p_{\theta_{\mathbf{z}}}(\mathbf{z}_t|\mathbf{z}_{t-1},\tilde{\mathbf{h}}_t)}{q_{\phi_{\mathbf{z}}}(\mathbf{z}_t|\mathbf{z}_{t-1},\tilde{\mathbf{g}}_t)} \Bigg] \\
= \mathbb{E}_{q_{\phi_{\mathbf{v}}}(\mathbf{v}|\mathbf{x},\mathbf{m},\mathbf{s},\mathbf{y})} \Bigg[& \log p_{\theta_{\mathbf{m}}}(\mathbf{m}|\mathbf{v},\mathbf{s},\mathbf{y}) \\
& + \sum_t \mathbb{E}_{q_{\phi_{\mathbf{z}}}(\mathbf{z}_{1:t}|\tilde{\mathbf{g}}_{1:t})} \Big[\log p_{\theta_{\mathbf{x}}}(\mathbf{x}_t|\mathbf{z}_t,\tilde{\mathbf{h}}_t,\mathbf{v},\mathbf{s},\mathbf{y}) \Big] \Bigg] \\
& - D_{\mathrm{KL}}(q_{\phi_{\mathbf{v}}}(\mathbf{v}|\mathbf{x},\mathbf{m},\mathbf{s},\mathbf{y}) \| p(\mathbf{v})) \\
& - \sum_t \mathbb{E}_{q_{\phi_{\mathbf{z}}}(\mathbf{z}_{1:t-1}|\tilde{\mathbf{g}}_{1:t-1})} \Big[D_{\mathrm{KL}}(q_{\phi_{\mathbf{z}}}(\mathbf{z}_t|\mathbf{z}_{t-1},\tilde{\mathbf{g}}_t) \| p_{\theta_{\mathbf{z}}}(\mathbf{z}_t|\mathbf{z}_{t-1},\tilde{\mathbf{h}}_t)) \Big]
\end{aligned}$$

.

S2.2 Network Details

The approximate posterior distribution $q_{\phi_{\mathbf{v}}}(\mathbf{v}|\mathbf{x}_{1:T},\mathbf{m}_{1:T},\mathbf{s},\mathbf{y})$ of the static latent variable $\mathbf{v}$ is parameterized by a 2-layer Multilayer Perceptron (MLP), as shown in Table A. Both RNNs for $\mathbf{h}$ and $\mathbf{g}$ are implemented as GRU cells with an input size of 64 and hidden dimension 128. Their inputs are given by linear transformations of $[\mathbf{x}_t,\mathbf{v}]$ and $[\mathbf{x}_t,\mathbf{h}_t,\mathbf{v}]$, respectively, followed by tanh. The dynamics models of the latent variable $\mathbf{z}_t$, both at inference and generation time, are implemented as 2-layer MLPs, presented in Table B. The generation of feature vectors $\mathbf{x}_t$ at each time step as well as the full sequence of missingness masks $\mathbf{m}_{1:T}$ are also implemented as MLPs, with details provided in Table C and Table D, respectively.

We use the Adam optimization algorithm [67] to update the networks of all models presented in this work. The choices of hyperparameters for each model can be found in Table E. For the dynamical VAE baselines (SRNN and KVAE), we largely used the implementations provided by Girin et al. [60], with some slight changes and extensions. The implementation of our model, as well as the experimental pipeline necessary to reproduce our results, can be found at `https://github.com/simonbing/HealthGen`.

Our experiments were conducted on a high-performance compute cluster, consisting mainly of NVIDIA V100 GPUs. In order to reproduce our results on a comparable hardware architecture, it would take approximately 200 GPU days.

S2 References

67. Kingma DP, Ba J. Adam: A Method for Stochastic Optimization. International Conference on Learning Representations. 2015.
