# Supplementary material for: Conditional generation of medical time series for extrapolation to underrepresented populations
Source: PLOS Digit Health. 2022 Jul 19;1(7):e0000074. doi: 10.1371/journal.pdig.0000074 (PMC9931259; doi:10.1371/journal.pdig.0000074)
Supplement: S3 Appendix — (PDF) [file pdig.0000074.s003.pdf]

S3 Data set characteristics

Table A: Descriptive statistics of the features included in the MIMIC-III data set. The value column shows the median value of the respective variable with the 10th and 90th percentiles indicated in parantheses. The right-most column shows the percentage of missing values for each input feature.

**Feature**	**Value**	**Miss.** [%]
Alanine aminotransferase	31.00 (12.00, 216.95)	98.198
Albumin	3.30 (2.30, 4.20)	98.710
Albumin ascites	1.45 (0.78, 3.03)	99.995
Albumin pleural	2.00 (1.10, 2.60)	99.996
Albumin urine	13.20 (1.40, 161.19)	99.994
Alkaline phosphate	84.00 (48.00, 205.00)	98.244
Anion gap	14.00 (10.00, 20.00)	94.942
Asparate aminotransferase	41.00 (17.00, 321.50)	98.197
Basophils	0.30 (0.10, 0.85)	98.533
Bicarbonate	24.00 (18.00, 28.00)	94.260
Bilirubin	0.70 (0.30, 3.50)	98.214
Blood urea nitrogen	18.00 (9.00, 50.00)	94.213
CO2	23.00 (17.00, 28.00)	98.515
CO2 (ETCO2, PCO2, etc.)	25.00 (20.00, 29.67)	92.637
Calcium	8.40 (7.20, 9.40)	96.178
Calcium ionized	1.14 (1.01, 1.28)	95.743
Calcium urine	2.40 (0.40, 13.08)	99.994
Cardiac Index	2.49 (1.82, 3.69)	98.056
Cardiac Output Thermodilution	4.90 (3.35, 7.20)	98.299
Cardiac Output fick	5.54 (3.70, 9.05)	99.707
Central Venous Pressure	11.00 (5.00, 17.00)	91.292
Chloride	105.00 (98.00, 112.00)	92.977
Chloride urine	47.00 (14.00, 116.60)	99.902
Cholesterol	164.00 (110.80, 226.20)	99.796
Cholesterol HDL	45.00 (30.00, 66.70)	99.834
Cholesterol LDL	93.00 (47.00, 147.00)	99.843
Creatinine	0.90 (0.60, 2.40)	94.201
Creatinine ascites	1.10 (0.56, 6.76)	99.998
Creatinine body fluid	1.10 (0.94, 1.66)	99.999
Creatinine pleural	1.20 (0.60, 3.25)	99.999
Creatinine urine	78.00 (24.00, 186.00)	99.736
Diastolic blood pressure	60.00 (44.50, 80.00)	68.667
Eosinophils	2.00 (1.00, 6.00)	99.981
Fibrinogen	220.00 (131.00, 434.00)	98.713
Fraction inspired oxygen	0.50 (0.40, 1.00)	98.022
Fraction inspired oxygen Set	0.50 (0.40, 1.00)	97.423
Glascow coma scale total	14.00 (3.00, 15.00)	94.824
Glucose	136.25 (95.00, 226.00)	88.420
Heart Rate	84.00 (63.00, 109.00)	68.040
Height	170.09 (154.97, 182.94)	99.440

Table A: (continued)

**Feature**	**Value**	**Miss. [%]**
Hematocrit	31.90 (24.10, 40.80)	91.177
Hemoglobin	10.80 (8.10, 13.90)	91.924
Lactate	2.00 (1.00, 4.88)	96.639
Lactate dehydrogenase	260.00 (156.00, 757.00)	99.228
Lactate dehydrogenase pleural	172.00 (65.20, 1485.00)	99.991
Lactic acid	2.10 (1.00, 5.10)	98.029
Lymphocytes	11.00 (3.80, 28.10)	98.061
Lymphocytes ascites	18.00 (2.00, 57.60)	99.982
Lymphocytes atypical	2.00 (1.00, 5.00)	99.874
Lymphocytes atypical CSL	1.50 (1.00, 3.00)	99.998
Lymphocytes body fluid	18.00 (2.00, 68.20)	99.973
Lymphocytes percent	13.00 (4.00, 39.00)	99.993
Lymphocytes pleural	30.00 (3.00, 79.40)	99.991
Magnesium	1.90 (1.50, 2.40)	95.863
Mean blood pressure	78.00 (61.00, 99.50)	68.884
Mean corpuscular hemoglobin	30.50 (27.50, 33.20)	95.043
Mean corpuscular hemoglobin concentration	34.10 (31.90, 35.80)	95.040
Mean corpuscular volume	89.00 (82.00, 97.00)	95.043
Monocytes	4.00 (1.70, 7.10)	98.081
Monocytes CSL	20.00 (4.00, 54.00)	99.966
Neutrophils	81.20 (60.00, 91.70)	98.050
Oxygen saturation	98.67 (94.00, 100.00)	67.963
Partial pressure of carbon dioxide	41.00 (32.00, 52.00)	92.637
Partial pressure of oxygen	195.00 (88.00, 387.00)	97.213
Partial thromboplastin time	30.60 (23.50, 55.50)	94.662
Peak inspiratory pressure	23.00 (14.00, 32.00)	97.442
Phosphate	3.50 (2.20, 5.30)	96.903
Phosphorous	3.40 (2.20, 5.20)	97.871
Plateau Pressure	18.00 (14.00, 25.00)	98.226
Platelets	203.00 (104.00, 352.00)	93.685
Positive end-expiratory pressure	5.00 (5.00, 10.00)	99.095
Positive end-expiratory pressure Set	5.00 (5.00, 8.00)	96.835
Post Void Residual	163.26 (65.40, 382.75)	99.955
Potassium	4.20 (3.40, 5.22)	91.172
Potassium serum	4.10 (3.40, 5.00)	98.676
Prothrombin time INR	1.30 (1.00, 2.00)	94.650
Prothrombin time PT	14.20 (12.20, 19.36)	94.652
Pulmonary Artery Pressure mean	26.00 (17.00, 40.00)	98.976
Pulmonary Artery Pressure systolic	33.00 (23.00, 47.00)	93.292
Pulmonary Capillary Wedge Pressure	16.00 (8.00, 27.00)	99.902
Red blood cell count	3.70 (2.75, 4.72)	95.039
Red blood cell count CSF	16.50 (1.00, 2950.00)	99.966
Red blood cell count ascites	1050.00 (43.20, 13450.00)	99.982
Red blood cell count pleural	3000.00 (182.60, 103866.40)	99.991
Red blood cell count urine	5.00 (1.00, 80.70)	99.701
Respiratory rate	17.00 (11.00, 25.00)	67.945

Table A: (continued)

**Feature**	**Value**	**Miss. [%]**
Respiratory rate Set	14.00 (10.00, 20.00)	97.250
Sodium	138.00 (133.00, 143.00)	92.165
Systemic Vascular Resistance	1037.04 (646.09, 1652.88)	98.060
Systolic blood pressure	117.00 (92.67, 150.00)	68.656
Temperature	36.56 (35.56, 37.61)	88.330
Tidal Volume Observed	564.00 (424.00, 800.00)	96.496
Tidal Volume Set	550.00 (450.00, 700.00)	97.480
Tidal Volume Spontaneous	460.00 (0.00, 714.00)	99.135
Total Protein	5.90 (4.83, 7.27)	99.990
Total Protein Urine	57.50 (13.00, 388.40)	99.967
Troponin-I	1.70 (0.30, 21.00)	99.914
Troponin-T	0.07 (0.01, 1.42)	98.723
Venous PvO2	43.00 (29.00, 71.00)	99.974
Weight	77.80 (55.50, 108.08)	97.047
White blood cell count	11.10 (5.70, 20.00)	94.052
White blood cell count urine	4.00 (1.00, 40.90)	99.706
pH	7.37 (7.27, 7.46)	92.402
pH urine	5.50 (5.00, 7.00)	98.473

Table B: Percentage of positive samples for the extracted labels, per subpopulation.

		`vent`	`vaso`	`colloid_bolus`	`crystalloid_bolus`	`niv`
	Overall	12.02%	10.30%	0.95%	9.61%	37.74%
Sex	Male	12.17%	10.53%	1.08%	9.94%	37.70%
	Female	11.84%	10.00%	0.79%	9.19%	37.79%
Age	<30	12.54%	10.25%	1.27%	8.48%	37.77%
	31-50	12.34%	10.17%	0.97%	9.64%	37.66%
	51-70	11.93%	10.52%	1.03%	10.26%	38.29%
	>70	11.90%	10.15%	0.82%	9.14%	37.24%
Ethnicity	White	11.82%	9.99%	0.88%	9.56%	37.82%
	Other	13.18%	11.25%	1.22%	10.11%	38.12%
	Black	11.51%	10.57%	1.27%	9.75%	37.08%
	Hispanic	11.79%	11.35%	0.53%	8.27%	36.68%
	Asian	12.71%	11.28%	0.83%	9.26%	36.46%
Insurance	Medicare	12.04%	10.11%	0.86%	9.32%	37.47%
	Private	11.93%	10.59%	1.13%	9.90%	38.00%
	Medicaid	12.29%	10.32%	0.90%	10.17%	38.57%
	Government	12.38%	10.95%	1.05%	9.52%	36.19%
	Self Pay	11.53%	9.22%	0.21%	10.48%	40.25%

Table C: Summary of the static variables for the extracted MIMIC-III patient cohort.

		Patients
Sex	Male	19,494 (57%)
	Female	14,978 (43%)
Age	<30	1,832 (5%)
	31-50	5,489 (16%)
	51-70	12,942 (38%)
	>70	14,209 (41%)
Ethnicity	White	24,643 (71%)
	Other	5,183 (15%)
	Black	2,667 (8%)
	Hispanic	1,137 (3%)
	Asian	842 (2%)
Insurance	Medicare	18,317 (53%)
	Private	11,846 (34%)
	Medicaid	2,782 (8%)
	Government	1,050 (3%)
	Self Pay	477 (1%)
