# Supplementary material for: Conditional generation of medical time series for extrapolation to underrepresented populations
Source: PLOS Digit Health. 2022 Jul 19;1(7):e0000074. doi: 10.1371/journal.pdig.0000074 (PMC9931259; doi:10.1371/journal.pdig.0000074)
Supplement: S4 Appendix — (PDF) [file pdig.0000074.s004.pdf]

S4 Samples of synthetically generated data

For the additional synthetic data visualization we preform a preselection of the generated patients based on lower than average missingness, in order to visualize samples where dense measurements over time are present and the dynamics are more evident . After this preselection, we randomly select three patients to visualize amongst them in Figs A, B and E. We also show two examples of randomly selected patients without this preselection in Figs C and D.

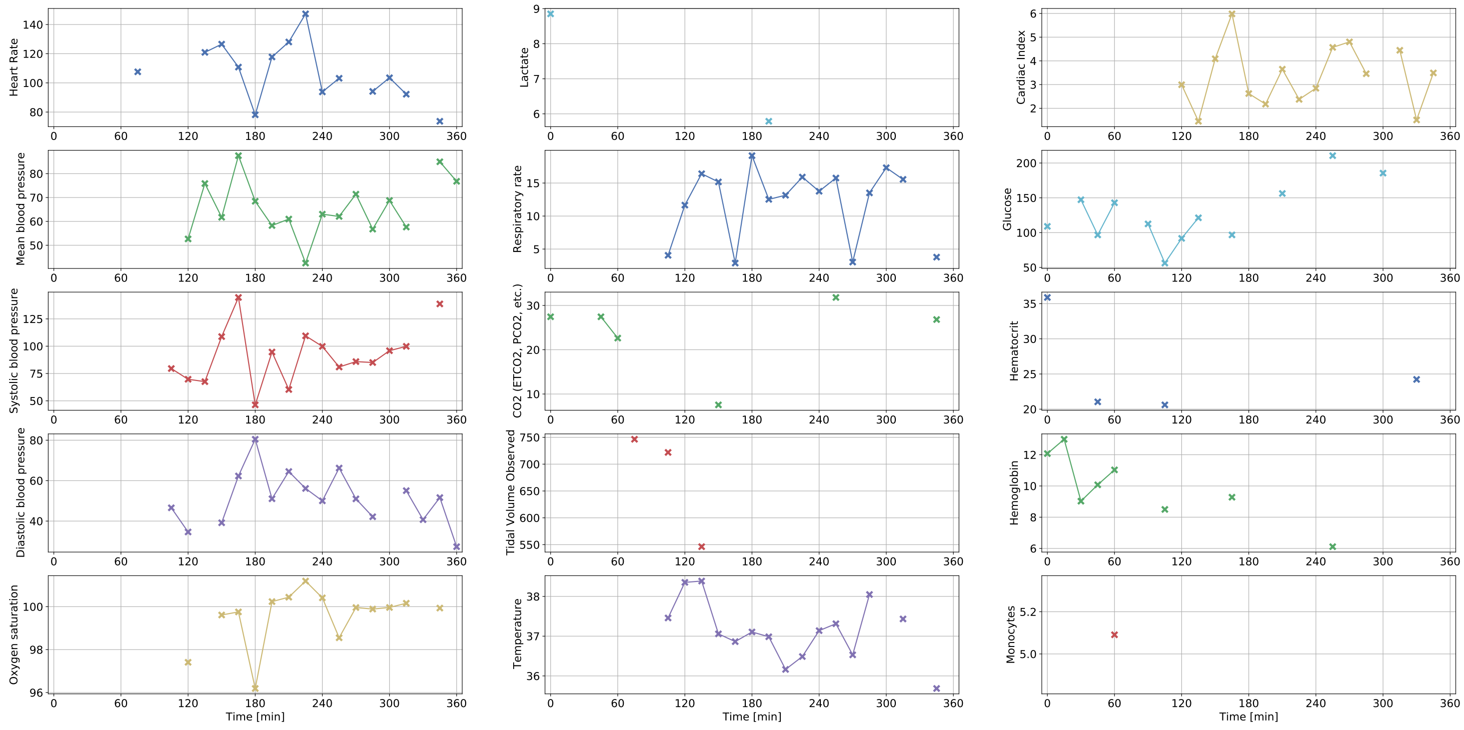

Figure A: Feature time series of a synthetically generated patient.

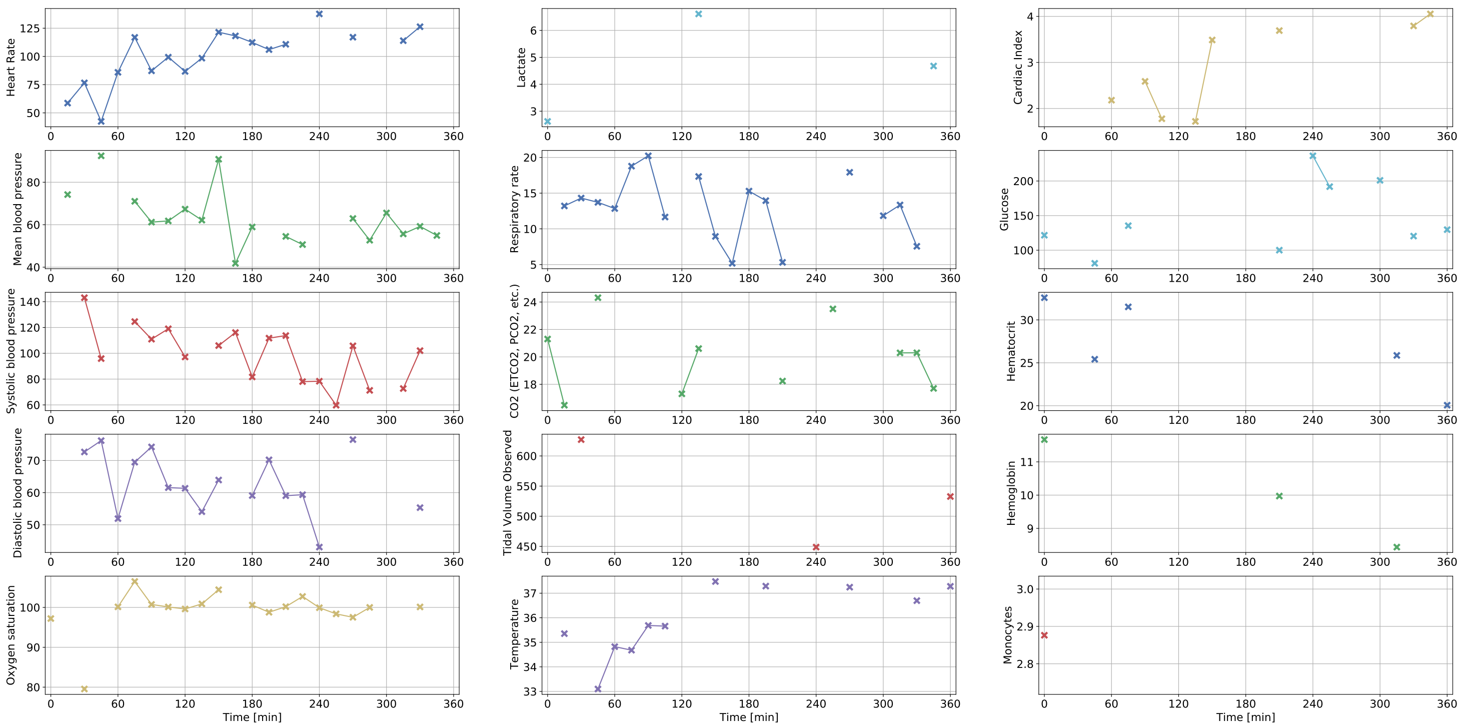

Figure B: Feature time series of a synthetically generated patient.

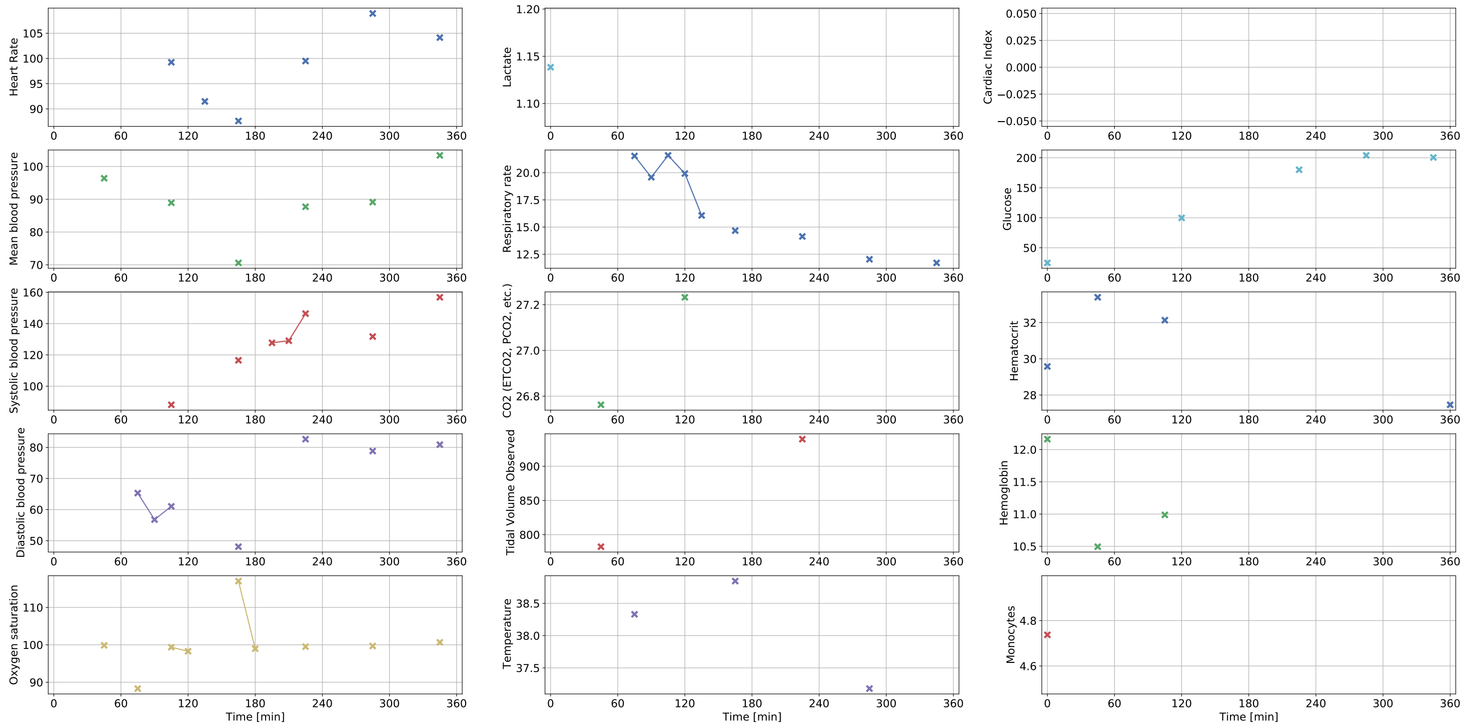

Figure C: Feature time series of a synthetically generated patient.

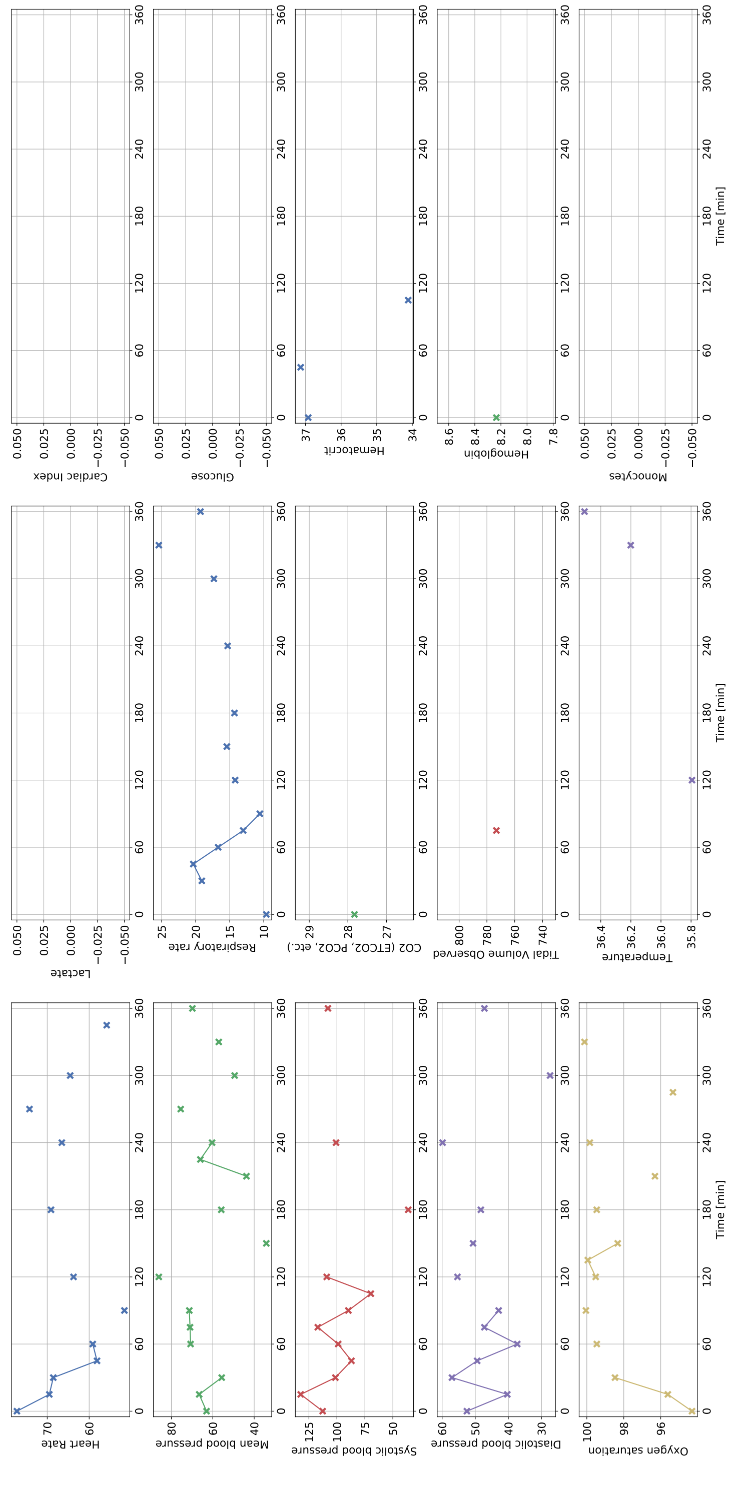

Figure D: Feature time series of a synthetically generated patient.

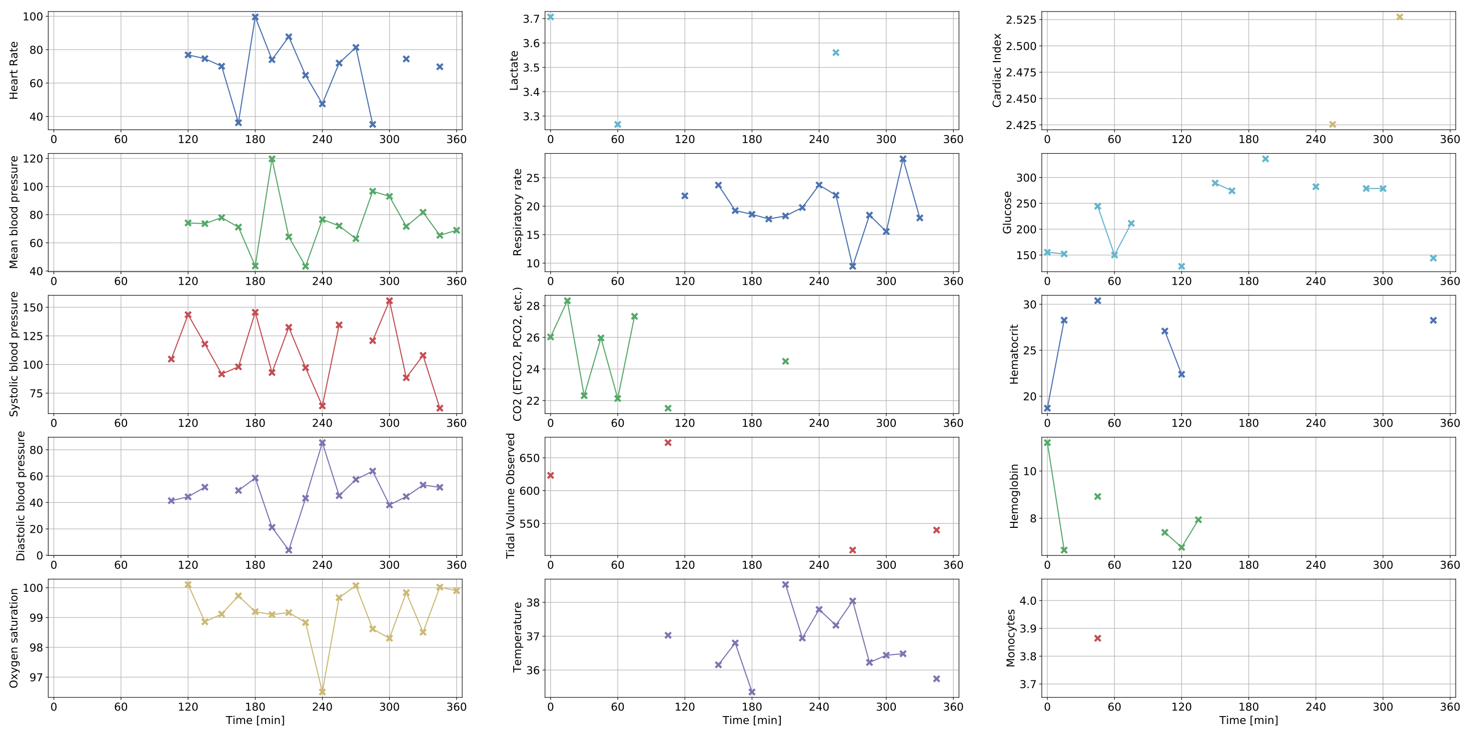

Figure E: Feature time series of a synthetically generated patient.-
