# Supplementary material for: Conditional generation of medical time series for extrapolation to underrepresented populations
Source: PLOS Digit Health. 2022 Jul 19;1(7):e0000074. doi: 10.1371/journal.pdig.0000074 (PMC9931259; doi:10.1371/journal.pdig.0000074)
Supplement: Attachment — Submitted filename: Response_Letter.pdf [file pdig.0000074.s005.pdf]

Response to the Editor and Reviewers

Dear Dr. Guillot, Dear Reviewers,

We thank you for your time and consideration spent on reviewing our manuscript. We are highly encouraged by your positive feedback regarding the merit of our presented research as well as your constructive criticism pointing out potential for improvement. In the following, please find our detailed response to your respectively raised concerns. A thoroughly edited and revised version of our manuscript, incorporating your feedback and reflecting the improvements proposed by your reviews, has been submitted. Thanks to your critical assessment of our work and your highly useful comments and questions, we believe that this updated manuscript meets the requirements for publication and presents innovative and relevant research to your readership.

Editor

Editor Comment — There is a need to bridge the gap between the current state of the manuscript and the journal's readership. In particular, the Material and Method section does a poor job in its current state: it contains a lot of acronyms and abbreviations, it make uses of many variables and distributions that are not defined, the rationale of the model is not stated anywhere. Equations and diagrams can not be a substitute to explanations in plain English. This section requires a thorough re-writing.

Reply: We thank you kindly for your feedback regarding the gap between our initial manuscript and the intended readership of the journal. Our goal is to present our findings to a broad, interdisciplinary audience. In light of this, the initial version of the methods section is perhaps too dense and technical and we have revised and re-written this section to provide a more gentle introduction to the basic approach of our model to an audience that is not familiar with these specialized methods. Figures and graphs have been supplemented with more descriptive captions in plain language and are supplemented with explanations in the text, which aim to invoke an intuitive understanding of our chosen approach and the model we present, without requiring expert knowledge beforehand. We hope that this revised section, as well as other, smaller changes in the manuscript strike a balance between technical details and intuitive understanding in order to properly address the diverse readership it is intended for.

Reviewer 1

Reviewer Point P 1.1 — Since the author is working on underrepresented population groups, it would be better to discuss some other works including Federate Learning, transfer learning [1, 2], population allocation [3], meta learning [4], etc. Also, it would be better to mention some bias reduction and mitigation methods [5].

[1] Wang, Z., Dai, Z., Póczos, B. and Carbonell, J., 2019. Characterizing and avoiding negative transfer. In Proceedings of the IEEE/CVF Conference on Computer Vision and Pattern Recognition (pp. 11293-11302).

[2] Gao, Y. and Cui, Y., 2020. Deep transfer learning for reducing health care disparities arising from biomedical data inequality. Nature communications, 11(1), pp.1-8.

[3] Rolf, E., Worledge, T.T., Recht, B. and Jordan, M., 2021, July. Representation Matters: Assessing the Importance of Subgroup Allocations in Training Data. In International Conference on Machine Learning (pp. 9040-9051). PMLR.

[4] Qiu, Y.L., Zheng, H., Devos, A., Selby, H. and Gevaert, O., 2020. A meta-learning approach for genomic survival analysis. Nature communications, 11(1), pp.1-11.

[5] Thompson, H.M., Sharma, B., Bhalla, S., Boley, R., McCluskey, C., Dligach, D., Churpek, M.M., Karnik, N.S. and Afshar, M., 2021. Bias and fairness assessment of a natural language processing opioid misuse classifier: detection and mitigation of electronic health record data disadvantages across racial subgroups. Journal of the American Medical Informatics Association, 28(11), pp.2393-2403.

Reply: Approaches other than synthetic data generation to increase the fairness of medical machine learning applications have indeed been brought forth and our initial manuscript did not reflect this in enough detail. Therefore, we have extended the introduction section with a paragraph citing these works and discussing how our approach may be seen to complement these methods. We thank the reviewer for pointing out this omission.

Reviewer Point P 1.2 — Another issue is the confounding factors [6,7]. It would be better to consider some factors, like age, sex, education background, ethnicity, etc. If you do not want to consider these confounding factors, please discuss the reasons.

[6] Ewers, R.M. and Didham, R.K., 2006. Confounding factors in the detection of species responses to habitat fragmentation. Biological reviews, 81(1), pp.117-142.

[7] Sul, J.H., Martin, L.S. and Eskin, E., 2018. Population structure in genetic studies: Confounding factors and mixed models. PLoS genetics, 14(12), p.e1007309.

Reply: Precisely as the reviewer points out, confounding factors play a critical role when modeling clinical populations. While we do not focus on these in the same level of detail as the works cited by the reviewer, we do address this topic to a certain degree. The limited setting which we investigate in this work is how time invariant demographic variables (age, sex, ethnicity, etc.) may influence the binary treatment variable (i.e. whether or not a treatment was required). This treatment variable has an effect on the dynamics of the generated time series of a patient (this binary variable is precisely what the downstream task aims to predict from the time series), but the demographic variables likely have a confounding effect. By explicitly including these demographic variables, our model aims to deal with this confounding and mitigate potential biases that follow, which we successfully demonstrate with our experiments.

Other variables such as educational background, as the reviewer suggests, likely also have an effect, but the variables we have included in this study are limited to those readily available in the raw data, which educational background unfortunately was not. A more principled understanding of confounding effects between static variables is indeed highly relevant, but was out of the scope of this work, as modeling such effects would likely require a more principled inclusion of such structural knowledge in the model. The approach we present here can be understood as a first proof of concept that certain confounding demographic factors can individually be included, and may serve as a basis for future development in this direction.

These points surrounding confounding factors have been added to the revised manuscript in the paragraph in the introduction section, added for the previous point P1.1. We thank the reviewer for this helpful comment.

Minor comments

Reviewer Point P 1.3 — The reference should be sorted in order. Reference [1] is not cited in the article.

Reply: We thank the reviewer for pointing out the citation inconsistency. We have fixed this in the revised version of the manuscript.

Reviewer 2

Reviewer Point P 2.1 — Figure 1, Figure 4, and Appendix D are used to support the argument that the synthetic time series are close, but not too close to the originals. However, as these results are purely qualitative, it's hard to tell if they are are generalizable. This argument would be much more compelling if the authors could propose an appropriate measure of similarity and to demonstrate that this measure showed appropriate characteristics over a large dataset.

This suggestion is made with full understanding that the appropriate metric is far from obvious, as the goal would be to measure success in hitting a "sweet spot" between complete lack of correlation and memorization. Possibilities such as distance correlation/covariance might be considered, but it is not clear that they would suffice. Additional challenges include the need to ensure that synthesized time series exhibit clinically appropriate and feasible behavior. For example, a synthetic dataset with high correlation to an actual dataset might still be inappropriate if the synthetic data exhibiting unrealistically large fluctuations. If a metric-based approach is not feasible, another alternative might be an adversarial approach - if an appropriately-trained classifier was not able to successfully classify sequences as either being real or synthetic, this might be seen as an argument for the success of the data synthesis approach.

Reply: Finding an appropriate metric is indeed one of the main challenges in synthetic data evaluation. We attempt to address this via our evaluation pipeline (described in Section 4.4) in order to define a quantitative metric, as the reviewer suggests. Regarding Figures 1 and 4, there seems to be a slight misconception as to what point these aim to make. Figure 4 especially only aims to make a visual point that the synthetic data is not merely and exact copy of the real data, nothing more. We have updated Figure 4's caption to clarify this. Figure 1 and the figures in Appendix S4 (previously Appendix D) are solely meant to provide a first indication that the synthesized data is at least visually similar to the real data, but also show that visual inspection alone does not suffice to measure its quality. Section 2.2 has been revised to clarify this intention.

The quantitative measure for how close we are to the characteristics of the real data is the comparison of the AUROC score when evaluating the downstream classifier on the real data vs. on the synthetic data. As we argue in the manuscript, this metric captures what is truly relevant for pragmatically useful synthetic data: can it be utilized to develop models that are later deployed on real data? We initially also considered moment matching or adversarial metrics, but would argue that these amount to contrived metrics that may only indirectly shed light on whether or not the clinically useful characteristics of the real data have been captured by the synthetically generated data. In fact, we believe that our evaluation based on the presented metric as opposed to previously utilized metrics constitutes a decisive advantage in terms of evaluation power with respect to previous approaches.

Minor comments

Reviewer Point P 2.2 — The discussion of the colloid bollus augmentation results in Figures 2 and 3 seems to understate the potential importance of those results. Tables C.2 and C.3 clearly illustrate that the number of government-funded colloiud bolus positive samples is very small, representing 0.95% (for colloid) and 3% (government funded). From the base of roughly 34,000 patients, this amounts to approximately 10 patients at the intersection of these two groups (assuming that the two factors are roughly independent). It's not surprising that the initial results are so poor, but the remarkable improvement on the augmented data is well-worth emphasizing.

Reply: We thank the reviewer for observing this remarkable result and are encouraged to stress this in Section 2.4 of the manuscript.

Reviewer Point P 2.3 — Regarding the presentation of the methods, the figures are useful and the equations are helpful. However, the details are a bit dense, reading as if presented for a NeurIPS audience, as opposed to the presumably broader audience that might be reading PLoS Digital Health. A slightly gentler introduction might be preferred for this audience.

Reply: This useful feedback was also raised by the editor and the methods section has accordingly been thoroughly rewritten to accommodate the broad readership of PLoS Digital Health better.

Reviewer Point P 2.4 — The lack of external validation might be seen as a weakness. Although there is no reason to believe that this method would not work on a second data set, this validation should be explored at some point in the future, and deserves mention in the current manuscript.

Reply: We considered using external validation data sets, and ideally would have liked to include them in this work, but their availability in a readily usable format was the key limiting factor. The MIMIC-III data set was chosen in an attempt to maximize the diversity of patients stemming from a single data set. While validation on external data would be ideal, the heterogeneity provided by the large number of patients in the MIMIC-III data, from different ICU units and treatment environments, recorded over a period of over ten years, is favourable to other, more homogeneous data sets. These points have additionally been emphasized in Section 3.4 in the revised manuscript.

Reviewer 3

Reviewer Point P 3.1 — The paper sets up 4 static variables and 5 classification tasks, but the authors only show results for a fraction of possible combinations of static variables and tasks. Additional information is needed to show that the model's performance holds for the other combinations of static variables and classification tasks.

Reply: As a preliminary experiment, we computed the scores for all possible combinations of static variables and downstream tasks for the real data. These results have been included in the updated manuscript in Appendix S1.4. Since performing full experimental runs (i.e. training all baselines and evaluating them) for all possible settings is heavily computationally demanding, we use the

aforementioned preliminary results and focus on those cases where a minority class underperforms and our model may therefore provide a benefit. For these cases we do full experimental runs. Out of these cases, we present those in the main text where our model can provide the biggest improvement, highlighting the settings where our model provides the greatest benefit. In Section 3, we discuss these settings and attempt to reason about which settings our model may be useful for and which settings are more challenging for its current state.

We agree that the results reported in the previous version of the manuscript may give the impression of testing a too limited number of setting and thus we have additionally conducted full experimental runs and included the full results of all other settings where our model might provide a benefit, in Appendix S1.4. As mentioned in the discussion section of the manuscript, these results show that cases where the scores of the respective subpopulations are closer together seem to be harder to improve upon with synthetic data generation. In total, we now perform the full experimental evaluation for 10 of the 20 possible combinations of static variables and tasks, in which almost all cases our model displays the most favourable performance.

We wish to stress again that we never claim that our model's performance holds generally for any possible combinations, but rather argue that it may provide a benefit for those cases where a minority group clearly underperforms with respect to the remaining population. We believe the benefit of our model's conditional generation capabilities in these settings to be substantial and the fact that it cannot improve upon the performance for any general combination of demographic group and downstream task does not diminish this.

Reviewer Point P 3.2 — In figure 4 the authors show a synthetic patient and data of the 3 closest real patients. While the data of the real patients looks similar, the synthetic data looks very different (different value ranges, different trends), leading to doubts whether their method of identifying nearest neighbors for synthetic patients is working. Additional statistics for i.e. a sample of 10 synthetic patients and the distance to their nearest neighbors, as well as the distance between the nearest neighbors would give more insights.

Reply: As the reviewer correctly points out, the real samples appear qualitatively closer to each other than the synthetic sample. However, we argue that this does not directly allow the conclusion to be drawn that the method to determine the nearest neighbors does not work as intended. Figure 4 is solely meant to provide a visual, qualitative inspection to see if the synthetic data is merely a direct copy of any real patients, not to determine whether or not the synthetic data captures the relevant characteristics of the real data. This is measured qualitatively via the evaluation score described in Section 4.4. Therefore, the synthetic data not being qualitatively too similar to the real data is in fact desirable. We stress that not only the visualized data points of the time series, but also the patterns where measurements are missing are important features of the time series. In light of this, we argue that the synthetic time series does display qualitatively similar characteristics with its nearest real neighbours. Another clear indication of the method to find the nearest neighbours working is the fact that the nearest real time series are in fact highly similar. To underline this, we present additional synthetic patients and their respective nearest neighbors, together with the distance between the synthetic sample and its real nearest neighbors in Appendix S1.6. These additional visualizations show that while qualitative similarities between the synthetic sample and its nearest real neighbours exist, they are never exact copies of each other. Furthermore, these additional samples show that the visualized real nearest neighbours of each respective sample are also qualitatively very similar to each other, underlining the proposed method to determine the

nearest neighbours to be working as intended.

As proposed by the reviewer, we also report the quantitative distances between the visualized samples. We stress however, that the reported distances only permit limited interpretability as the utilized cosine distance does not scale linearly. As other reviewers raised similar concerns with respect to Figure 4, we have updated the corresponding section in the manuscript to clarify this for future readers.

Reviewer Point P 3.3 — As described in P 3.2, Figure 4 leads to doubts whether their method of identifying nearest neighbors works. The authors should therefore consider using another baseline approach like dynamic time warping to measure the distances between patient time series to verify that their method is working.

Reply: As mentioned in the previous reply, we are highly confident in originally utilized method working, underlined clearly by the real nearest neighbors being very similar. As suggested by the reviewer, we perform the same nearest neighbour analysis using dynamic time warping (cf. Figure R1), but found that the resulting nearest neighbours were qualitatively closer together using the original method (cf. Figure 4, manuscript). This could perhaps be due to dynamic time warping not being able to capture the important binary patterns of missingness, which the original method can, as it compares the latent embeddings of the time series, where the patterns of missingness are encoded as well.

Reviewer Point P 3.4 — For results 2.4 they make an unfair comparison: "Since the baselines cannot generate data conditioned on static variables, we have them unconditionally generate the same number of overall samples that our model augments the real data with." As the baseline models can create any number of synthetic patients, they should create synthetic patients (and discard synthetic patients from overrepresented classes) until they have enough to create a balanced dataset. This shows that the model is able to create underrepresented groups with the same quality as the other approaches, while also showing that their conditional approach makes this much simpler and more targeted.

Reply: While this approach to compare our model to the baselines would be intriguing to test, we stress that the baselines' limitations make this fundamentally impossible. The baseline approaches cannot conditionally generate patients based on demographic variables, which also means that the synthetic data they generate carries no information about these demographic groups whatsoever. The labels to indicate group membership of patients do not exist in the data the baselines synthesize, therefore we could not discard generated patients in a targeted fashion to arrive at a balanced data set. Our model's capability to generate these patients in a targeted fashion is a decisive advantage over the baselines.

Perhaps the fact that we can still report scores per subpopulation for the baselines, despite their generated data not carrying the group labels, causes confusion. This is possible for any input data, since the final evaluation score is computed on the held out real data, which always contains the labels indicating group membership and thereby allowing the evaluation metric to be reported per group.

Reviewer Point P 3.5 — The downstream classification model's strong performance on some underrepresented groups indicates that group membership might already carry information. Some initial

information on how the medical interventions are distributed over group memberships would help put the results better in context.

Reply: We thank the reviewer for the useful suggestion and have updated Table S3.2 to reflect how the interventions are distributed across all subpopulations. Given this additional information, we see that group membership and positive labels can be assumed to be independent. We would like to stress that even if there were an evident correlation between the two, the downstream classifier could not use this information directly. The downstream classification model has no access to any of the labels indicating group membership, it must perform its classification given only the input time series.

Reviewer Point P 3.6 — P.6 "In all considered tasks, our approach significantly outperforms the state-of-the-art models in synthetically generating medical time series." This is not true. See for example Table 1 (improvement for colloid_bolus likely not significant), of Fig.3b overall (performance worse than baseline, significant difference).

Reply: This statement was only intended in reference to the experiments described in this section (2.2) and the original wording has been changed to clarify this. The improvement for the colloid_bolus task is indeed not significant. We thank the reviewer for pointing out this unfortunate oversight and have reformulated this sentence to better reflect this.

Reviewer Point P 3.7 — Very small underrepresented groups see a large increase in predictive power of the downstream model. This could be because the generative model learns to 'overfit' on

Reply: We argue that the increase in predictive power of the downstream model for previously underrepresented groups is not due to overfitting of our model to this one group, as this would imply that other subpopulation groups' performance would subsequently decrease. In our results, we show the fact that the increase in performance for one subpopulation does not occur at the cost of any other group's performance. Furthermore, overfitting would be noticeable in the reported evaluation scores, as these are computed on the held out validation set that is not utilized at any other step. From our reported results, this cannot be observed.

Reviewer Point P 3.8 — The authors define a way to measure fairness, and claim that their approach produces fairer results, but they never provide numerical evidence to support this.

Reply: We initially believed the visual presentation of our experimental results surrounding fairness would suffice to convey the benefit our model provides, leading to omit the numerical results of these experiments. We agree with the reviewer that this omission is possibly misleading, as we do introduce a definition of how we measure increased fairness. To avoid any confusion, we have added the numerical results supporting our claims that our model's generated data leads to more fair downstream results, as well as the formula for the metric we utilize. This information as been added to the revised manuscript in Appendix S1.3. We have additionally clarified our initial description of how we measure fairness in the introduction section.

Reviewer Point P 3.9 — If the model overfits on underrepresented groups, it is also likely to reproduce bias found in the dataset. "Furthermore, the quality and diversity of the generated data

which our model produces is limited by the real data with which it is trained" is not enough of a warning for this possibility.

Reply: To a certain degree our model surely reproduces certain biases seen in the training data, as this is a fundamental issue any machine learning model faces. However, we argue that we demonstrate that our model's conditional generation capability helps to overcome issues of biases connected to the static variables we condition on. While our model is evidently better equipped to deal with biases stemming from known attributes than the considered baselines, other biases may of course be reproduced. We have updated Section 3.4 in the manuscript to clarify this point.

Reviewer Point P 3.10 — Ideally, one would use more than one dataset for evaluation to ensure that the model actually learns to generalize the properties of underrepresented groups and not overfits on patterns observed in the data. However, I concede that this is very difficult due to the limited availability of medical datasets and their limited compatibility.

Reply: We agree that this is a very valid point and would have also liked to include an external validation data set. However, as the reviewer points out, this is highly challenging. Given the limited availability of suitable data sets in this space, we opted to base our study around the MIMIC-III data set, in an attempt to maximize the diversity of the data available from a single source. The MIMIC-III data consists of a large number of patients (>30k) from a wide range of different ICU units, treatment environments and a time span of over ten years, resulting in a data set considerably more heterogeneous than others with less patient data, collected in a single environment. As this point was raised by another reviewer as well, we comment on this in the manuscript in Section 3.4.

Reviewer Point P 3.11 — The term missingness is mentioned in the introduction (p.3). While in the later context it becomes clear what missingness means, but a short explanation would improve reading flow here.

Reply: We thank the reviewer for this suggestion, this has been reformulated in the introduction to clarify what we mean with missingness.

Reviewer Point P 3.12 — The initial description of the dataset should explicitly mention that it contains only patients from one hospital, i.e. "[. . .] patients that spent time in the intensive care unit (ICU) in the Beth Israel hospital . . . ". Together with the preceding introduction, this can otherwise make it look like the dataset contains a variety of patients from different populations and the HealthGen model can be used out-of-the-box by anyone wishing to improve their dataset diversity.

Reply: This has been clarified in Section 2.1 in the revised manuscript.

Reviewer Point P 3.13 — For Fig. 1 and Fig. 4 it is not clear why / how these features were selected for display from the set of all features. Reducing number of features and number of patients so the image can fit horizontally would immensely improve readability.

Reply: These features have been selected to visualize a representative subset of features which display the characteristic dynamics of a typical patient. Since we can already only show a fraction of all 104 features, maximizing the number of presented features resulted in a horizontal image in

the initial manuscript. We agree that this impairs readability, and will adjust these figures so that they can be presented horizontally in the final manuscript.

Reviewer Point P 3.14 — The benefit of having Fig. 1 is unclear, the displayed synthetic timeseries look very different from the real one, only the patterns of missingness seem appropriate.

Reply: Figure 1 is meant to at least once visualize some of the generated time series in the main text of the manuscript. The fact that the synthetic time series look different from the real ones based on visual inspection does not take away from quantitative results we present. We would argue that this figure underlines a point we aim to stress in the paper: visual inspection alone clearly does not suffice to determine the quality of the generated data. This figure in no way aims to convince the reader visually that our generated data is of high quality, we rather do this by means of our quantitative evaluation scheme. The manuscript has been updated in Section 2.2 to stress this and prevent any misunderstandings of this figure's intention.

Figures

Point P3.3

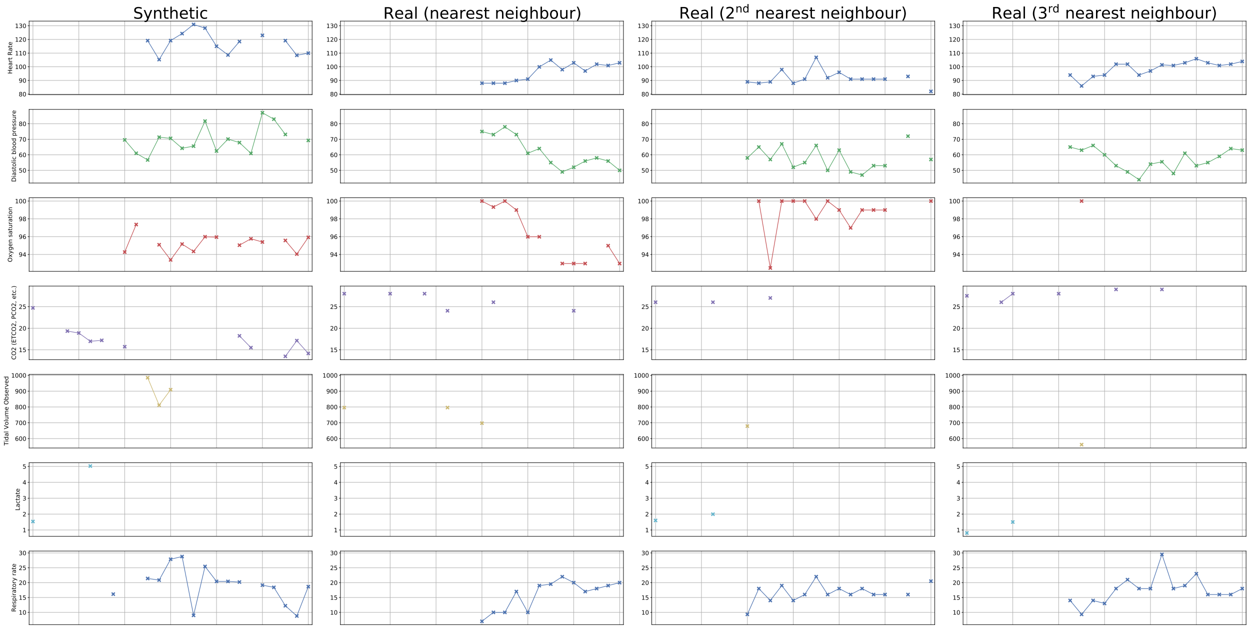

Figure R1: Synthetically generated patient (same as in the manuscript) and its nearest real neighbours, calculated using dynamic time warping.
